# The Past, the Present, and the Future: A Bibliometric Analysis of Failed/Fragile/Collapsed State Research During 1990–2020

**DOI:** 10.3389/frma.2022.720882

**Published:** 2022-02-04

**Authors:** Chi Swian Wong

**Affiliations:** Department of Political Science, Nanjing University, Nanjing, China

**Keywords:** data analysis, bibliometric review, science mapping, emerging trends, political economy, failed/fragile/collapsed state research, academic structure

## Abstract

The “failed/fragile/collapsed state” refers to state authority's complete or partial collapse, such as Somalia and Bosnia. According to Fragile States Index 2020 annual report, approximately 116 countries among 178 countries were in warning or alerting state quo, which hurts three-quarters of the world's population. A systematic scientometric interpretation of failed/fragile/collapsed state analysis would be helpful but is presently absent in the academic community. This review makes three donations by evaluating the 2,417 articles published in the Web of Science (WoS) Social Science Citation Index (SSCI) Collection between 1990 and 2020. First, it provides a unique prospect in failed/fragile/collapsed state studies through a detailed, systematic, and objective analysis. Second, the author has quantitatively tracked the progression of failed/fragile/collapsed state studies from 1990 to 2020. Finally, the author associated evolutionary trajectory analysis with future research directions, offering new pathways for failed/fragile/collapsed state studies. It also helps novice “failed/fragile/collapsed state” researchers and veteran scholars identify future research trends.

## Introduction

The “failed/fragile/collapsed state” refers to state authority's complete or partial collapse, such as Somalia and Bosnia (King and Zeng, 2001). According to Fragile States Index 2020 annual report delivered by the Fund for Peace (Messner de Latour, 2020), ~116 countries among 178 countries are in warning or alerting state quo, which hurts three-quarters of the world's population.

A growing number of studies on failed/fragile/collapsed states have been published, as seen in the following **Figure 4**. Some reviews have summed up these studies. Firstly, Brooks (2005) sought to challenge a global order and policy theoretical hypothesis, contending that the remaining state-based international framework failed to promote adequate responses to nation failure. Secondly, Di John (2010) provided a crucial analysis of later research that had sought to interpret what a “failed state” is and revealed why such states emerged. Thirdly, Nay (2013) disputed that the analytical underpinning of the state “fragility” and “failure” and concluded that the theories of “failed and fragile states” are deceptive, shallow, as well as policy-aligned tags that are volatile. Finally, Ferreira (2017) reviewed existing approaches to operationalize the failed/fragile/collapsed state concept.

In short, previous reviews sparked arguments and offered a foundational understanding of the field of failed/fragile/collapsed state studies since many years ago. However, recent years' rapid growth of failed/fragile/collapsed state studies means a thorough review of this academic field is more meaningful than decades before.

Unfortunately, the aforementioned current reviews focused only on a few articles published in political or economic. To the deepest of the author's understanding, no reviews have utilized the bibliometrics approach to scrutinize this domain's advancements comprehensively. As a result, we need a systematic and complete explanation of the academic development pathways, status quos, and future study directions. This review would systematically and technologically analyze the co-authorship structures among different nations, institutes, journals, and scholars, the hotspots, and the roadmap for future failed/fragile/collapsed state studies.

Second, the author has quantitatively tracked the progression of failed/fragile/collapsed state studies from 1990 to 2020. Specifically, this article shows how the frontiers of failed/fragile/collapsed state research shift yearly and gives readers a fast understanding of the academic structure of the substantially increasing group of failed/fragile/collapsed state research. Based on the 2417 samples of failed/fragile/collapsed state studies, this article blends landscape and timeline visualization to systematically examine failed/fragile/collapsed state literature.

Finally, the author associated evolutionary trajectory analysis with future research directions, offering new pathways for failed/fragile/collapsed state studies. In particular, the author has identified four possible research trends in failed/fragile/collapsed state studies:

- The definitions of the “failed state” and “fragile state” as well as measurement approaches of state failure.
- Systematically factors attribute to the state failure or state fragility from micro and macro levels.
- Multiple negative changes or catastrophic consequences that unequivocally triggered by state failure or state collapse; and
- Proposals encourage good governance and resist further conflicts and war within failed/fragile/collapsed states.

For each of the primary research trends proposed, the author recommends some scientific inquiries. In summary, this review catalyzes future failed/fragile/collapsed state studies by presenting academics with a comprehensive interpretation of the scientific groups, academic structure, hotspots, and future evolutions in the failed/fragile/collapsed state research domain.

## Theoretical Concepts

### Failed State

Although the term “failed state” was created during the 1990s, there is still no precise definition because of various research views after the Cold War. However, there still have been many mainstream classifications.

According to Longley (2020), the term “failed state” has no widely accepted meaning due to its subjective existence. Like “grace,” “fragility” is in the beholder's feeling. Suppose a state cannot enforce its principles steadily or present its inhabitants with essential profits and benefits. In that case, it is generally deemed “failed.” Revolution, rampant crime, ineffective and inflexible bureaucracies, nepotism, legislative incapacity, and military involvement in politics are common reasons for a state's failure/fragility/collapse.

Professors Bøås and Jennings both have criticized the term's ambiguity, claiming that heightened confusion in the wake of the September 11 attack, as the consequence of the war on terrorism, developed countries, in particular, regard “failed states” as a danger to international peace (Bøås and Jennings, 2005). However, such a viewpoint is too political and founded on a misconception of the state's genuine essence of failure. Otherwise, they suggest that a better vital question to consider is not whether the state fell but “To whom the state fails and how.”

### Fragile State

The “fragile state” is an academic topic that has become fashionable in the mid-90s since the September 11 terrorist attacks and has gained more momentum. Many policymakers and scholars conclude that the root of the contemporary conflict is within states rather than between them (Rapoport, 2001; Ahmad, 2002; Esses et al., 2002; Kellner, 2002; McInnes, 2003; Skitka et al., 2004; Murphy, 2005). Low-capacity and low-income countries in the developing world are projected to face acute risks to the economies of their western neighbors (Patrick, 2011). According to this logic, fragile states need economic development to offer security and essential goods to their people, lowering susceptibility and increasing resilience to inner and foreign challenges (Patrick, 2011). Fragile states, in this sense, share much of the same problems with failed states but on a much smaller scale. Their fragility foreshadows what would happen if their administrative procedures were not improved (François and Sud, 2006).

A fragile state has untrustworthy governments. According to Tyagi, fragile states are challenging to identify since they do not collect detailed crime and education statistics (Tyagi, 2012). Fragile states are primarily described as:

1) Conflict/post-conflict/crisis/war or political change circumstances with dynamics.
2) The status of the government is deteriorating.
3) The fact that domestic GDP growth is slow.
4) Long-term diplomatic or economic instability situations or deadlocks.

Fragile states are more vulnerable to crises in one or more sub-systems (Kornprobst, 2002). It is a country prone to internal and external disturbances as well as domestic and foreign disputes (Jackson and Rosberg, 1982). To allow policymakers to act correspondingly, fragile countries are measured in terms of their vulnerability, fragility, and risk (Call, 2011).

### State Collapse

The breakdown, failure, or collapse of a state is called “state collapse,” which is the total breakdown of a sovereign regime (Milliken and Krause, 2002). The increase of the state's disintegration after the Cold War—the dissolution of the regime and severe disturbance of justice and order in those separates of the world—is directly linked to the disturbance about a nation-state's destiny (Dragović-Soso and Cohen, 2008). State collapse is also seen as the ultimate type of state decline: “in countries where authority and domestic order have practically vanished, the dramatics of the decline of state authority are identified (Schachter, 1998).” Michael Reisman adds, “The collapsed state created more challenges than any other phenomenon about the prospect of the nation-state” (Reisman, 1997).

## Software Selection and Literature Search Method

### Software Selection

Although many software programs facilitate bibliometric analysis (Muñoz et al., 2020), many of them do not aid researchers in following the suggested workflow (Muñoz et al., 2020). Among them, VOSviewer (van Eck and Waltman, 2010), CiteSpace (Chen, 2006), Carrot2 (Osiński and Weiss, 2005), and Biblioshiny are the most useful tools.

Van Eck and Waltman created VOSviewer, a free Java tool for evaluating and displaying citation networks in the scientific collection. VOSviewer can construct and visualize bibliometric networks (van Eck and Waltman, 2013). The operation interface of VOSviewer 1.6.16 can be seen in Figure S1 in the Supplementary Material.

CiteSpace is a free Java program to view and analyze scientific literature patterns and trends (Chen, 2006). CiteSpace was used to list leading nations, institutes, specific journals, and scholars (Chen and Song, 2019; Chen et al., 2019). The structures created by The operation interface of CiteSpace 5.7.R4 can be seen in Figure S2 in the Supplementary Material.

Carrot2 is a free-source research results clustering engine (Stefanowski and Weiss, 2003). It is also created in Java and presented under the BSD license (Stefanowski and Weiss, 2003). The operation interface of Carrot2 4.3.1 can be observed in Figure S3 in the Supplementary Material.

Biblioshiny is an R language software serving quantitative analysis in scientometric and bibliometrics (Pritchard, 1969). The author utilizes Biblioshiny to display the top 5 journals' annual occurrence growth. The operation interface of Biblioshiny 3.1 can be seen in Figure S4 in the Supplementary Material.

**Figure 1:**
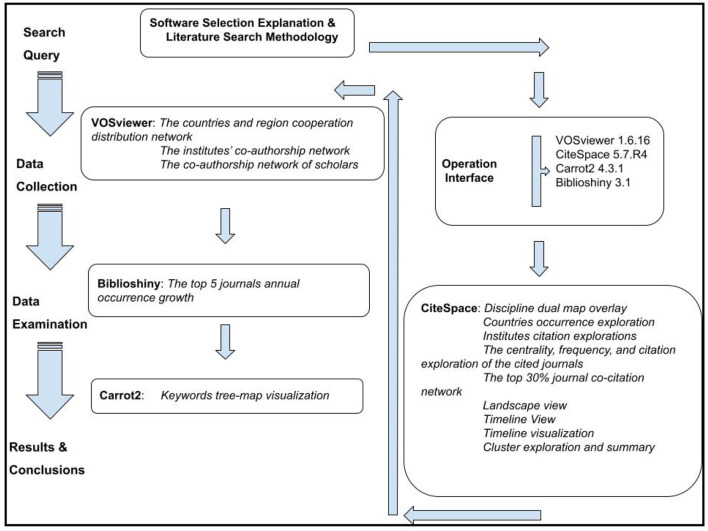
The flowchart of this review (by Google Draw).

The academic literature provides crucial details about scientific knowledge (Chen and Song, 2019). Citation-based exploration has also been adopted to systematically analyze the academic domain's evolutionary trends and intellectual framework. Figure 1 details this review's workflow.

### Literature Search Methodology

The author adopted the data collection procedures of Chen (2017). First, the author compiled a selection of query words based on a thorough scan of current review papers. Then, running a search query to examine whether the applied words from the WoS SSCI dataset could be found. The author compiled a list of candidate phrases utilizing text processing analysis to evaluate the pilot query effects.

The author replicated the above procedures several times until the search query had obtained and verified a reasonably convergent result. The ultimate detail of terms in this research is displayed in Table 1. The year 1990 was chosen as the starting time (After the drastic changes in Eastern Europe). The deadline is 2020/12/31 (The full-year nearest to the beginning of this research). The documents' “abstract,” “title,” “keyword plus,” and “author keywords” were all included in the WoS Core Collection's “TS” (Topic).

**Table 1:** Search queries for SSCI articles of failed/fragile/collapsed state research.

**Steps**	**Set**	**Search strategies**	**No. of papers**
First	# 1	(TS = (“fragile state*” or “failed state*” or “state* failure*” or “state* fragilit*” or “weak state*” or “collaps* state*” or “state* collaps*” or “breakdown state*” or “state* breakdown” or “break* state*” or “state* break*” or “crisis state*” or “state* crisis*” or “mafia state*” or “post-conflict state*” or “post conflict state*” or “war-torn state*” or “shadow state*”)) and language: (English) and document types: (article or review) Indexes=SSCI; Timespan=1990–2020	1,760
Second	# 2	(TS = (“fragile nation*” or “failed nation*” or “nation* failure*” or “nation* fragilit*” or “weak nation*” or “collaps* nation*” or “nation* collaps*” or “breakdown nation*” or “nation* breakdown” or “break* nation*” or “nation* break*” or “crisis nation*” or “nation* crisis*” or “mafia nation*” or “post-conflict nation*” or “post conflict nation*” or “war-torn nation*” or “shadow nation*”)) and language: (English) and document types: (article or review) Indexes=SSCI; Timespan=1990–2020	324
Third	# 3	(TS = (“fragile countr*” or “failed countr*” or “countr* failure*” or “countr* fragilit*” or “weak countr*” or “collaps* countr*” or “countr* collaps*” or “breakdown countr*” or “countr* breakdown” or “break* countr*” or “countr* break*” or “crisis countr*” or “countr* crisis*” or “mafia countr*” or “post-conflict countr*” or “post conflict countr*” or “war-torn countr*” or “shadow countr*”)) and language: (English) and document types: (article or review) Indexes=SSCI; Timespan=1990–2020	365
Fourth	# 4	#1 or #2 or #3	2417

*In WOS, “TS” = topic, relates to keywords, abstract, title and keywords plus in this field.*

*Various terms (such as weak states, shadow states, post-conflict states, mafia states, crisis states, state collapse, and so on) are interchangeable with “failed/fragile/collapsed states.” Besides, “nation” and “country” are interchaeable with “state”*.

As an unfortunate byproduct of collecting enough related literature as necessary, noise does not influence the ultimate results. This data collection approach coincides with the spirit of Chen et al. (2019). As shown in Table 1, since choosing “English” as the language as well as “Article” or “Review” as the form of publication for my research on failed/fragile/collapsed states, the author received 1,760 articles for “failed/fragile/collapsed state” research, 324 papers for “failed/fragile/collapsing nation” research, 365 papers for “failed/fragile/collapsing country” research and 2,417 papers for the consolidated strings of studies. The author has included 38,835 secondary documents cited by the 2,417 articles.

## Results

### Discipline Co-occurrence Analysis

**Figure 2:**
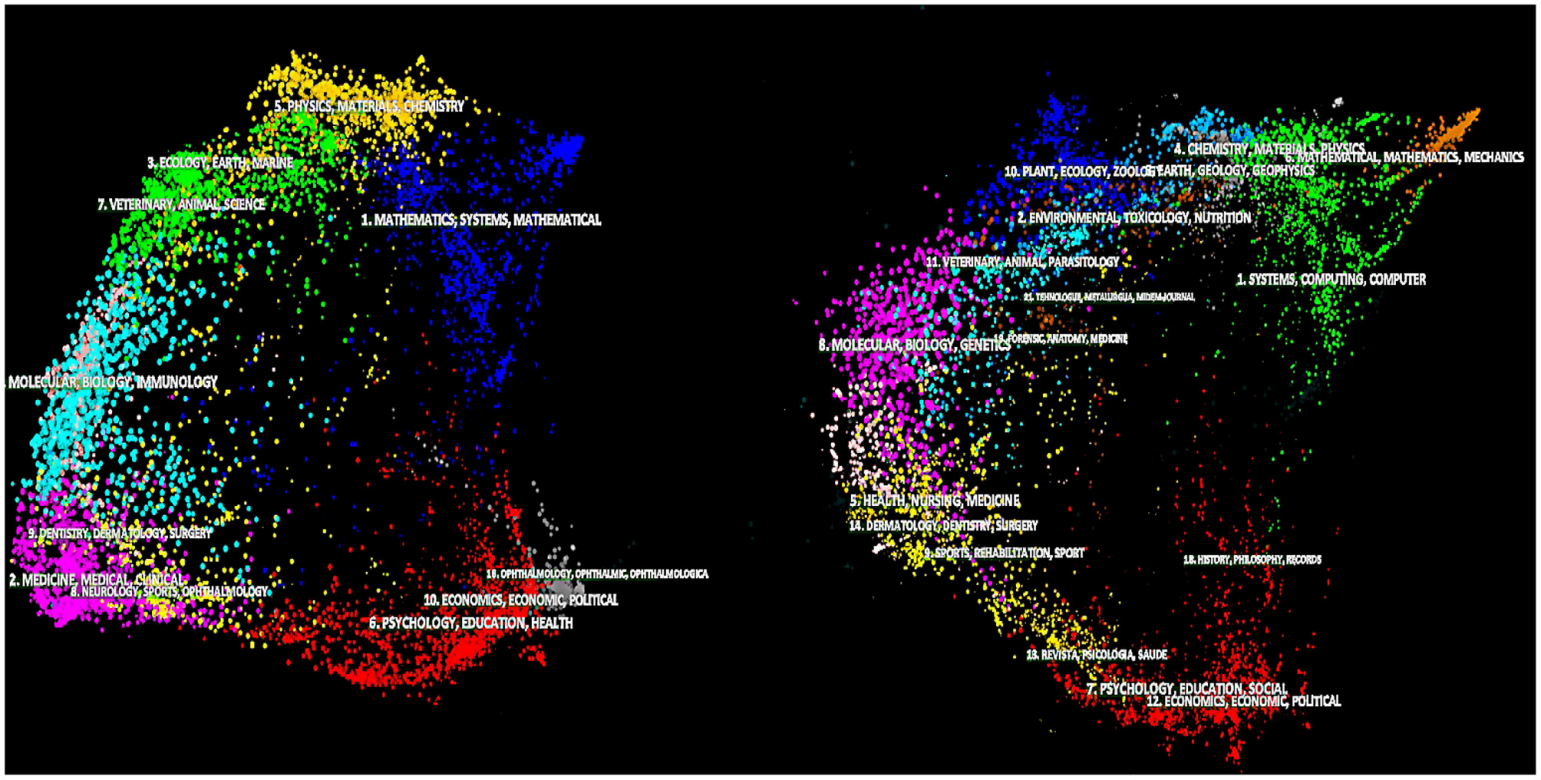
The CiteSpace 5.7.R4 interface.

**Figure 3:**
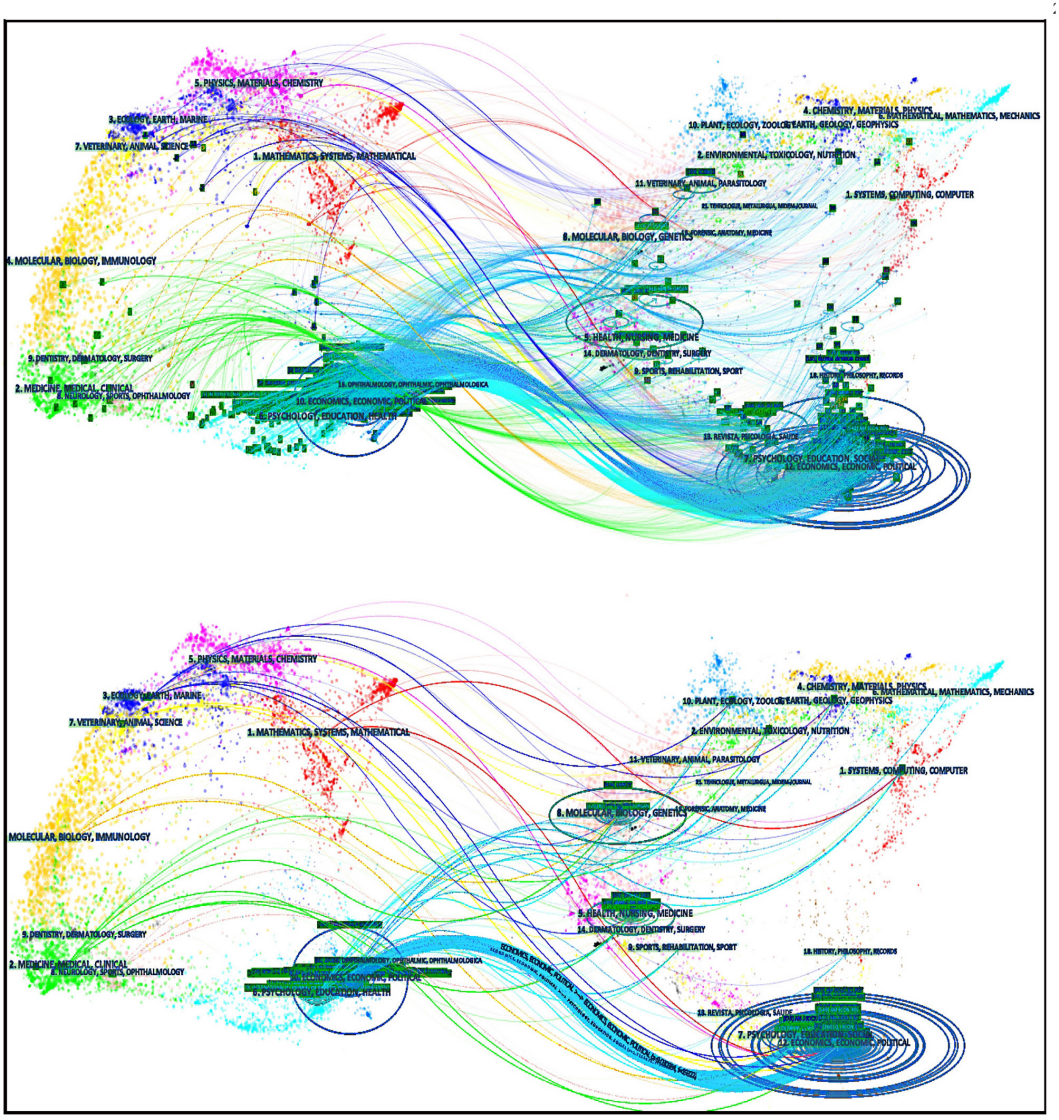
The journal–based dual–map of the Resource Curses, Dutch Diseases, and Conflict Resources–related papers on the global scientific mapping (by CiteSpace 5.7.R4).

Figure 2 depicts a CiteSpace display with annotations. It compares and contrasts the citing and cited map data. On the left is shown its referencing network diagram, which includes 10,330 referring journals. Blondel's clustering method identified the groupings in various colors (Blondel et al., 2008). On the right is shown its referenced graph, which includes 10,253 cited journals. CiteSpace's overall setup includes two kinds of styles: curves and arcs. In the arc style, a referral link is shown as a metaphorical arc. A citation link is represented as a smooth curve that travels from the originating journal to the citation's destination journal in the arc style. This arc style is meant to make the presentation of a large number of reference links easier to understand. In this failed state/fragile state/collapsed state research domain, dual–map overlay evaluations allowed tracing the conceptual basis of exceptionally fruitful and frequently cited articles. Figure 3 depicts the results.

The viewing of dual–map overlays was conducted using CiteSpace (Chen, 2006). Carley and his collaborators developed a journal–based dual–map overlay (Carley et al., 2017). It allowed displaying the publications in a particular data collection on the journal map of the global research domains. This review followed the articles in those journals' reference lists, overlaid them on another journal overlay graph, and connected the two graphs. Labeled ovals signify clusters of periodicals commonly referenced and cited.

Figure 3's upper section simplifies more details by focusing on referenced article clusters. This was done by modifying the thickness of the lines according to the density of citations, using the z–record of citation connections (Kim et al., 2016). Figure 3 reveals that failed state/fragile state/collapsed state-related publications are primarily distributed in the “economy, economic, and political” groups of journals. The cited articles, which can be considered the scientific domain's conceptual foundation, are mainly contained in the journal group “economy, economic, and political.” Figure 3 (lower half) depicts the major journal classes and their relationships, with the z–score used to scale the line thicknesses.

It can be illustrated that all the referring groups have cited articles from the “economics, economic, and political” or “psychology, education, and health” journal areas. It means that the academic foundation of the failed state/fragile state/collapsed state-related science remains relatively narrowly centered across particular scientific sub-domains.

### The Yearly Distribution

From 1990 to 2020, Figure 4 illustrates the results of failed/fragile/collapsed state/nation/country research records (May 13, 2021). It can be divided into three steps. Stage I (1990–2001), the annual articles rarely exceeded 22. There were a few exceptions in 1996 and 2000, mainly due to “failed/fragile/collapsed state” literature rather than the other two strings of literature. In Stage II (2002–2013), following the September 11 attacks, the number steadily raised from 6 articles in 1991 to 59 in 2002. Stage III (2014–present) has seen a significant rise in related studies, with 169 papers published in a single year in 2014 and 204 in 2020.

**Figure 4:**
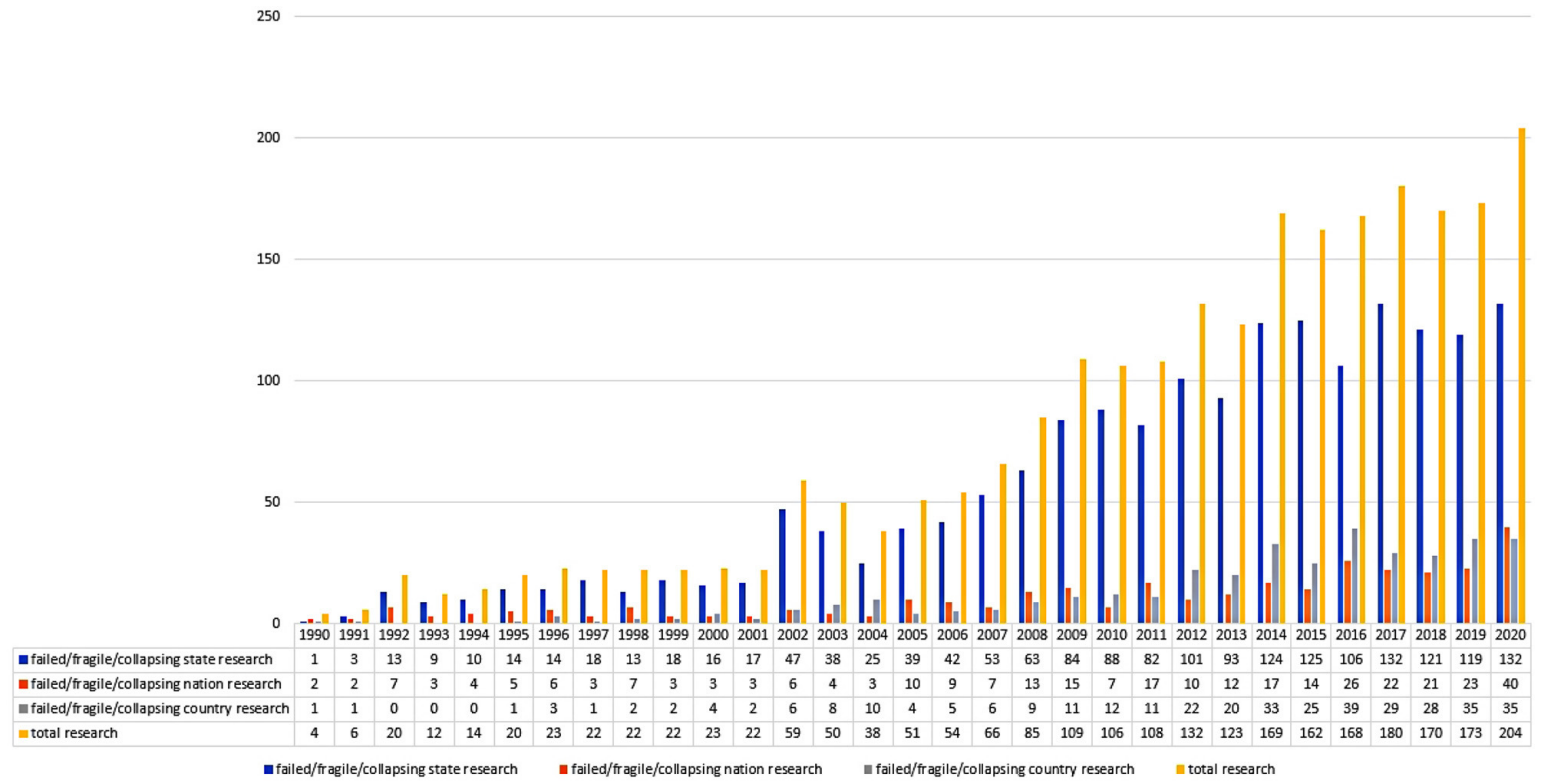
The annual distribution of three strings of articles from 1990 to 2020 (as of May 13, 2021).

Although it is tempting to believe that research on failed/fragile/collapsed state/nation/country research domain has grown in popularity, this shift can be recognized in the light of broader scholarly publication trends. It is unclear if the improved failed/fragile/collapsed state/nation/country research–related study outputs reflect this domain's increased academic importance if these phenomena are a function of increased relevance in political economy or sociology, or whether trends result from internal academic dynamics.

### Country Distribution and Regional Cooperation

The betweenness centrality measure of Freeman et al. (1979) identifies possible paradigm transition crucial moments across time. A node's centrality is a graph-theoretical attribute that measures how important the position of a node is in a net. Table S1 in the Supplementary Material shows that the United States and the UK played critical roles in the international cooperation network.

**Figure 5:**
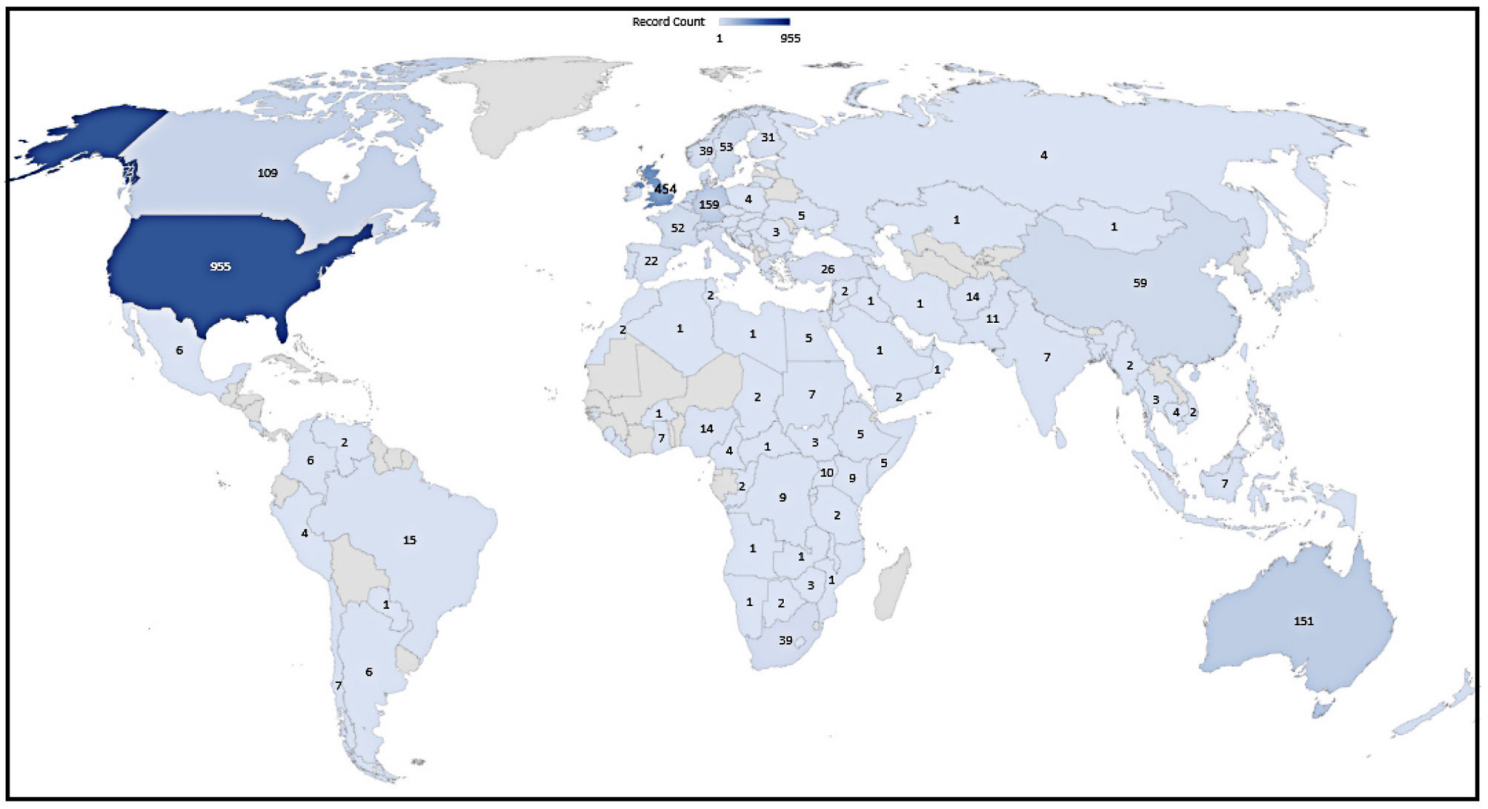
Different countries with publications.

The number of papers, Australia, Germany, Canada, the Netherlands, Switzerland, Sweden, France, and China, was ranked 3rd to 10th, as shown in Figure 5. However, Table S1 in the Supplementary Material shows that despite having the ninth-most publications, Sweden has a small betweenness centrality (0.05), advising that Sweden has not established close cooperation structures with diverse countries despite its many articles. France was ranked 8th in many articles, and it had the 5th highest betweenness centrality score, indicating that French intellectuals collaborated with academics from other countries more closely. Another phenomenon that cannot be ignored is that the US, Australia, the UK links are unsurprising given more significant academic trends. European and American nations played more critical positions in failed/fragile/collapsed state research's academic output and international cooperation.

In order to focus on the most critical clusters and filter out redundant information, the authors have intentionally limited the number of nodes when operating VOSviewer to as many as possible in the range of 20-40. Using VOSviewer, and the “minimal number of articles for a country” was set to 10. Thus, the network of Figure S5 in the Supplementary Material contains 37 of the total 117 countries/districts. By utilizing VOSviewer, for the number of documents, the USA ranked 1st and the UK ranked 2nd. Using CiteSpace, the results of countries with citation explosions are illustrated in Figure S6 in the Supplementary Material.

Citation explosion indicates a very active field of study, as seen in Figure S6 by CiteSpace. Citation explosions identify an explosive event that may span many years or just one year. A citation explosion shows that a certain publication is linked to increased citations. Kleinberg's technique identifies explosions in CiteSpace (Chen, 2006). The citation explosions of Israel last the most extended years, lasting 16 years (1993–2009), while the USA's exploration has the most explorations score (18.48).

The evolution of research on “failed/fragile/collapsed state” from the 1990s to lately 2000s relied mainly on scholars from the United States. England and Canada. Sweden has been rapidly strengthening in later periods, and its explosions are yet underway.

### Institutes' Distribution and International Cooperation

Once the “minimum number of institute papers” is set to 15 using VOSviewer, the author gains the institute's co-authorship framework. Of the 1,695 institutes, 25 meet the thresholds. Table S2 and Figure S7 in the Supplementary Material show that the remaining 25 institutes are divided into 10 clusters by VOSviewer. The University of Oxford has the most (38) documents, and Stanford has the most significant citation numbers (3,657). The rest of the top five institutions of publications are Columbia University (36), Harvard University (33), the University of London (30), and the University of Birmingham (24). The top five institutes of citation numbers are Stanford University (3,657), Columbia University (2,107), Harvard University (1,213), University of Oxford (942), and University of Manchester (899). All of them came from either the United States or the United Kingdom. Each of the top 25 academic institutions is from countries in Europe and North America. The West still dominates this research field.

By detecting the institutes with occurrence exploration using CiteSpace, the author can determine which institutes are picking up fast in this field. As illustrated in Figure S8 in the Supplementary Material, the explosions of Harvard University and University Nashville have relatively more considerable intensity (6.65 and 4.69). In contrast, Columbia University and Harvard University have the most prolonged duration (5 and 7 years). The frequency can be found in Table S3 in the Supplementary Material.

It is helpful to discuss how very productive academics shape the reputation of their institutes. As shown in Table 2, Tables S4, S5 and Figure S9 in the Supplementary Material using CiteSpace, the total citation numbers of James D. Fearon and David D. Laitinare are 3,342, which were half of the UK and much higher than Germany. James D. Fearon and David D. Laitinare can rank 3rd among countries regarding the total citation numbers. This phenomenon is similar for prominent institutions; for example, Stanford University can rank third of the total citation numbers, rivaling most countries. Productive academics shape the reputation of their institutes and vice versa.

**Table 2:** Citation number by countries using CiteSpace 5.7.R4.

**Rank**	**Country**	**Citations**	**Documents**	**Rank**	**Country**	**Citations**	**Documents**	**Rank**	**Country**	**Citations**	**Documents**
1	USA	24,895	955	11	Norway	767	39	21	Turkey	323	25
2	England	8,507	454	12	Scotland	634	46	22	Chile	299	6
3	Germany	2,559	159	13	Belgium	604	33	23	Brazil	298	15
4	Australia	1,787	151	14	Peoples R China	583	49	24	Spain	279	22
5	Switzerland	1,587	70	15	Greece	504	16	25	Wales	244	15
6	Canada	1,563	109	16	Finland	470	31	26	Uganda	235	10
7	Netherlands	1,348	83	17	Singapore	452	20	27	South Korea	220	34
8	Italy	1,171	48	18	South Africa	421	39	28	Kenya	210	10
9	Sweden	1,034	53	19	Denmark	420	44	29	Austria	201	14
10	France	851	52	20	Israel	388	31	30	Pakistan	195	13

### The Distribution and International Cooperation Between Scholars

The author uses VOSviewer to explore how authors collaborate for failed/fragile/collapsed state research. Only 30 of the 4,355 contributors match the requirements. They are represented in the ultimate networks in Figure S10 in the Supplementary Material since the “minimum number of citations for an author” is 120 and the “minimum number of documents for an author” is 2. As shown in Table S8 in the Supplementary Material, we can see that James D. Fearon and David D. Laitin are the researchers with the most (3,342) citation numbers and in the remarkable focus of the graph. James D. Fearon is a fellow at Stanford University's School of Humanities and Sciences, lecturer of political science, and a senior fellow for International Studies. His research focuses mainly on armed conflict and political disorder. Fearon's research activities include local and ethnic war, tribal disturbance, the politics of industrial outcome, and democratic accountability (James Fearon, 2021). David D. Laitin is also a political science professor at Stanford University. It can be seen that Egbert Sondorp, the researcher with the most (7) publications, is a professor at the Royal tropical institute in the Netherlands. His academic interests are mainly in health in fragile and conflict-affected areas.

Using CiteSpace to examine the top 50% of publications with the most citations each year from 1990 to 2020, the author detects no authors with citation explorations, which gives information that no specific article is linked with a roaring of citations. Collaboration among researchers is critical to advancing an academic field (Li et al., 2017). Figure S10 in the Supplementary Material reveals a network of academics with coordination.

### Cited Journals' Distribution

The author obtains the journal co-citation network via CiteSpace, as shown in Figure S11 in the Supplementary Material. The years per slice is 1. The selection criteria are top 30%, and the maximum number of selected items per slice is 100. Minimum spanning tree as well as the merged pruning network was the method. Larger nodes in Figure S11 in the Supplementary Material reflect higher citation occurrences. As shown in Table 3 and Tables S6, S7 in the Supplementary Material, the citation frequency of the journal from the American Political Science Review was 412. International Organization had a citation frequency of 340, and Third World Quarterly had a citation frequency of 339. With a citation frequency of 329, World Politics was classified fourth, accompanied by International Security (327), Journal of Peace Research (311), World Development (294), and Journal of Conflict Resolution (290). The academic subjects of failed/fragile/collapsed state research are primarily from politics and economics, based on the category details.

**Table 3:** The cited journals with frequency (by CiteSpace 5.7.R4).

**Rank**	**Freq**	**Cited journals**	**Rank**	**Freq**	**Cited journals**
1	412	American Political Science Review	11	244	Foreign Affairs
2	340	International organization	12	221	American Journal of Political Science
3	339	Third World Quarterly	13	205	Development and Change
4	329	World Politics	14	195	African Affairs
5	327	International Security	15	173	The New York Times
6	311	Journal of Peace Research	16	170	The Lancet
7	294	World Development	17	168	Comparative Political Studies
8	290	Journal of Conflict Resolution	18	158	The Quarterly Journal of Economics
9	288	American Economic Review	19	153	Journal of Political Economy
10	246	International Studies Quarterly	20	149	Journal of Modern African Studies

Using CiteSpace, we can see that the top 20 journals in terms of betweenness centrality score were almost all from politics and economics (Table 3), mainly including Theory International Politics (1.22), World Politics (1.04), American Political Science Review (0.82), and International Organization (0.82), confirmed the critical role of these journals in failed/fragile/collapsed state research. However, regarding exploration scores, Foreign Affairs was first with an exploration score of 33.63 in 2002–2011, followed by Thesis with an exploration score of 25.68 in 2018–2020. Other scientific journals with high exploration scores include World Politics, International Security, and American Political Science Review. It means that failed/fragile/collapsed state research is prevalent in politics, economics, sociology and has mushroomed in recent years.

### Journals Annual Occurrence Growth

This part was carried out using Biblioshiny, an R package for co-citation and bibliometric analysis (Muñoz et al., 2020). R is an ecological system, so all functionalities are available to users in an inclusive environment. Compared to most free software (e.g., CiteSpace and VOSviewer), Biblioshiny does not focus only on the data visualization but also on the correctness and statistical completeness of the results (Muñoz et al., 2020).

Figure S12 in the Supplementary Material illustrates the top 5 journals' annual occurrence growth by Biblioshiny. The author applies the loess smoothing procedure. The smooth line is displayed using locally weighted smoothing and regression test—loess smoothing assists in the apprehension of treads over the year (Nasir et al., 2020). After 1998, annual occurrences of Third World Quarterly, which is the principal origin of failed/fragile/collapsed state research literature, have expanded considerably. From 2013 until now, there has been a downward tendency of annual occurrences in Third World Quarterly, Journal of Peace Research, Development and Chance. From 2013 onwards, World Development, Journal of Conflict Resolution has a steady rise trend. Scientists and directors can examine these journals during pandemics since they may have a valuable understanding of the socio-economic effects. When regressing through ten years, most of the journals have no apparent loess smoothing.

## The Intellectual Framework of Failed/Fragile/Collapsed State Research

### Landscape View by CiteSpace

The creation of a fresh scientific field must draw on the conceptual basis of diverse related disciplines. Academic journal papers reflect the groundbreaking of special domains, and some publications in those papers serve as a scientific foundation for them (Li et al., 2017). The structure of co-citation evaluation is a dynamic approach to reliably characterize and visualize the academic foundation's function (Zhu et al., 2019). The author, therefore, accepted a co-citation structure evaluation to investigate the academic framework of failed/fragile/collapsed state research. CiteSpace was used to investigate the connections between cited sources that make up the failed/fragile/collapsed state field's intellectual foundation. The following picture of the landscape is based on articles from 1990 to 2020 (Figure S13 in the Supplementary Material). The selection criteria are g-index, and the scale factor k = 25, creating a structure of references cited yearly. The labeling source was “Title,” and the clustering process was performed using the log-likelihood rate approach. Aiming to increase the co-citation network's clarity, the author chose pathfinder and pruning sliced networks, which retain the essential connections (Wang et al., 2018). The network's modularity score is 0.3406 and cannot be regarded as highly, suggesting that failed/fragile/collapsed state research items are not fully occupied in cluster co-citations (Chen et al., 2019). The structure has a high median silhouette score of 0.9249, meaning that each cluster's publication is remarkably persistent for quality (Li et al., 2017). All important cluster scores over 0.7 are reasonable, explaining that the failed/fragile/collapsed state science mapping is high-quality cluster analysis. Each node's size represents the citation frequency obtained from the related references.

### Timeline View by CiteSpace

**Figure 6:**
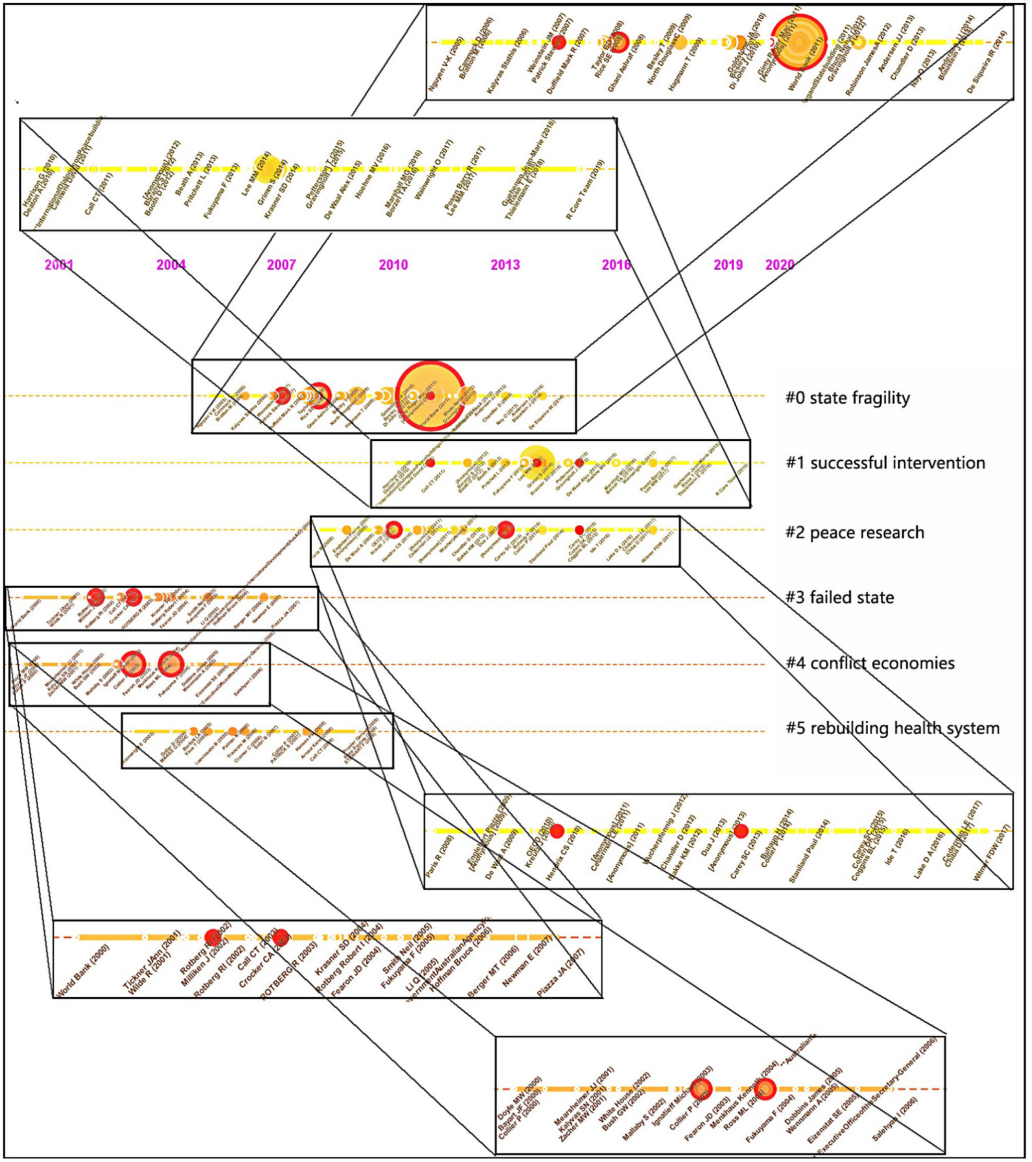
The co-citation network of timeline view (by CiteSpace 5.7.R4).

As shown in Figure 6, CiteSpace was used again to provide a timeline view structure of co-citations to examine the primary literature on the evolution point to reflect each cluster's development trajectory and status quo (Figure 6). The author chose the g-index as the selection criterion (scale factor k = 25). The author chose pathfinder and pruning sliced networks, which retain the essential connections (Wang et al., 2018).

Because of the length limitations of this research, the author focused on clusters 0–3; which were the top four major clusters. The Carrot2 tool was used to explore them further.

### Leading Clusters Explanations

**Figure 7:**
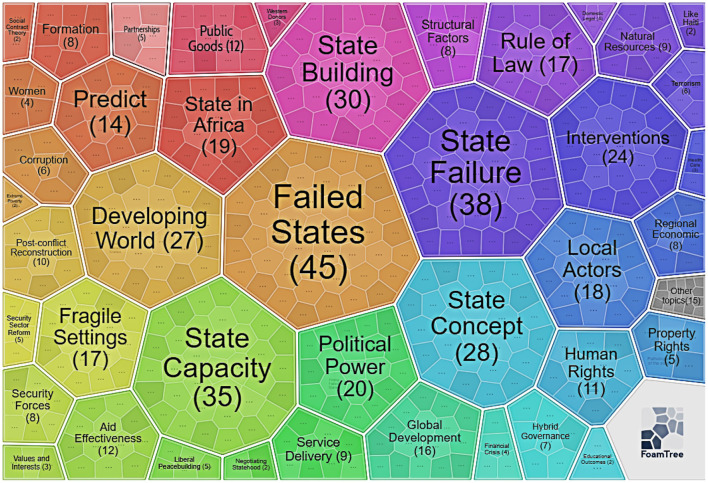
Keywords in Cluster 0 (by Carrot2 4.3.1).

**Figure 8:**
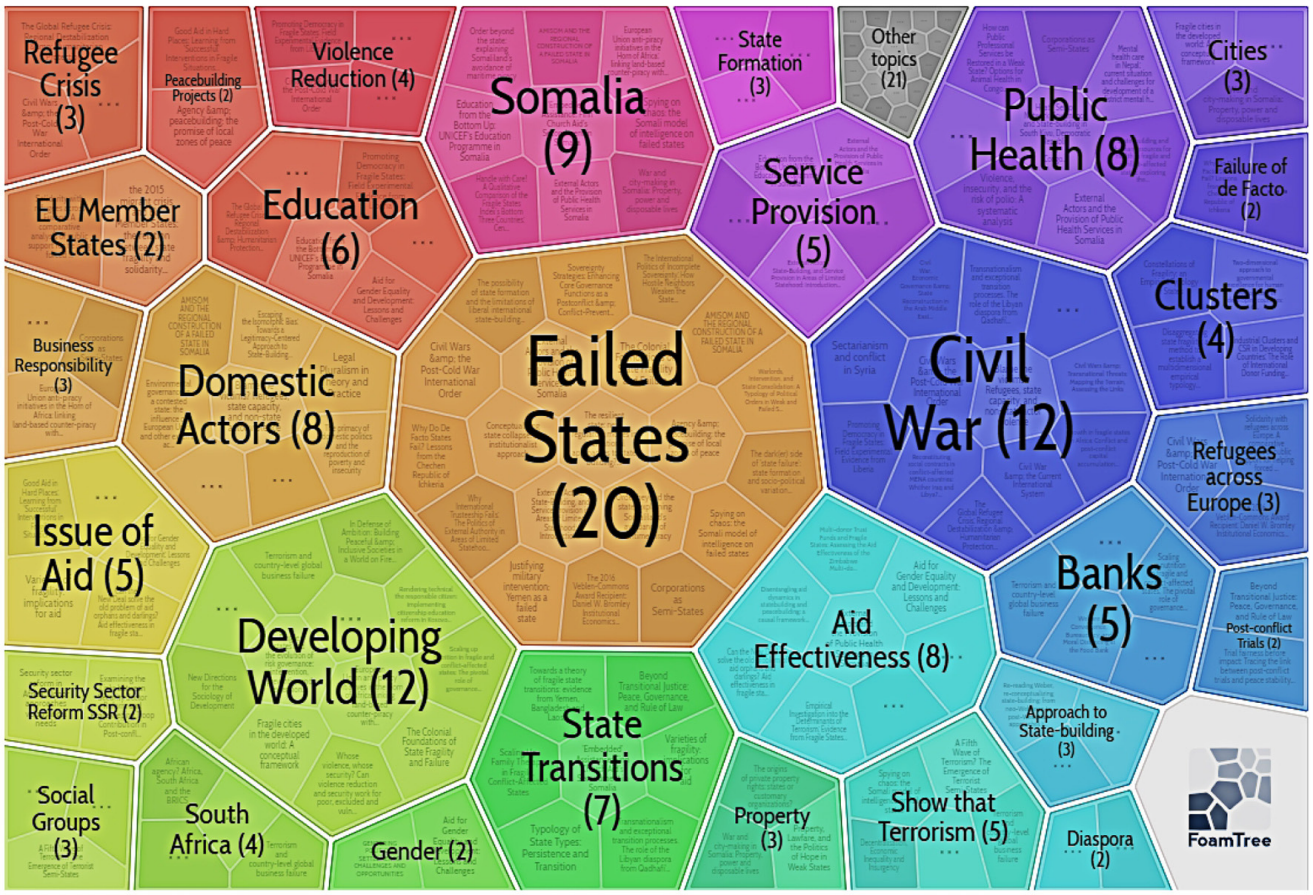
Keywords in Cluster 1 (by Carrot2 4.3.1).

**Figure 9:**
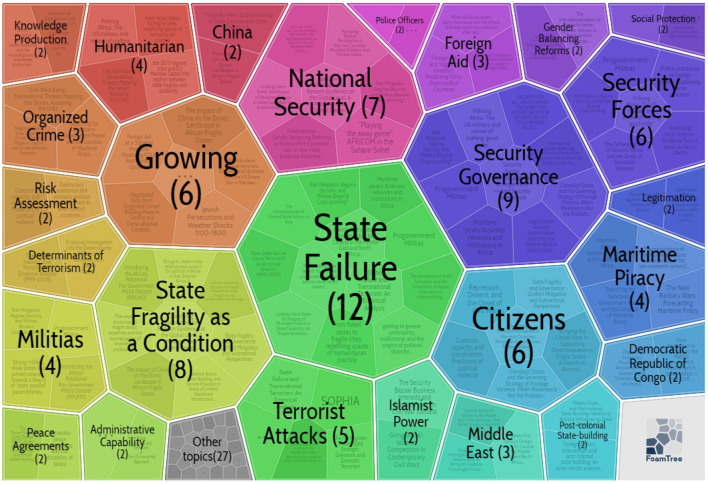
Keywords in Cluster 2 (by Carrot2 4.3.1).

**Figure 10:**
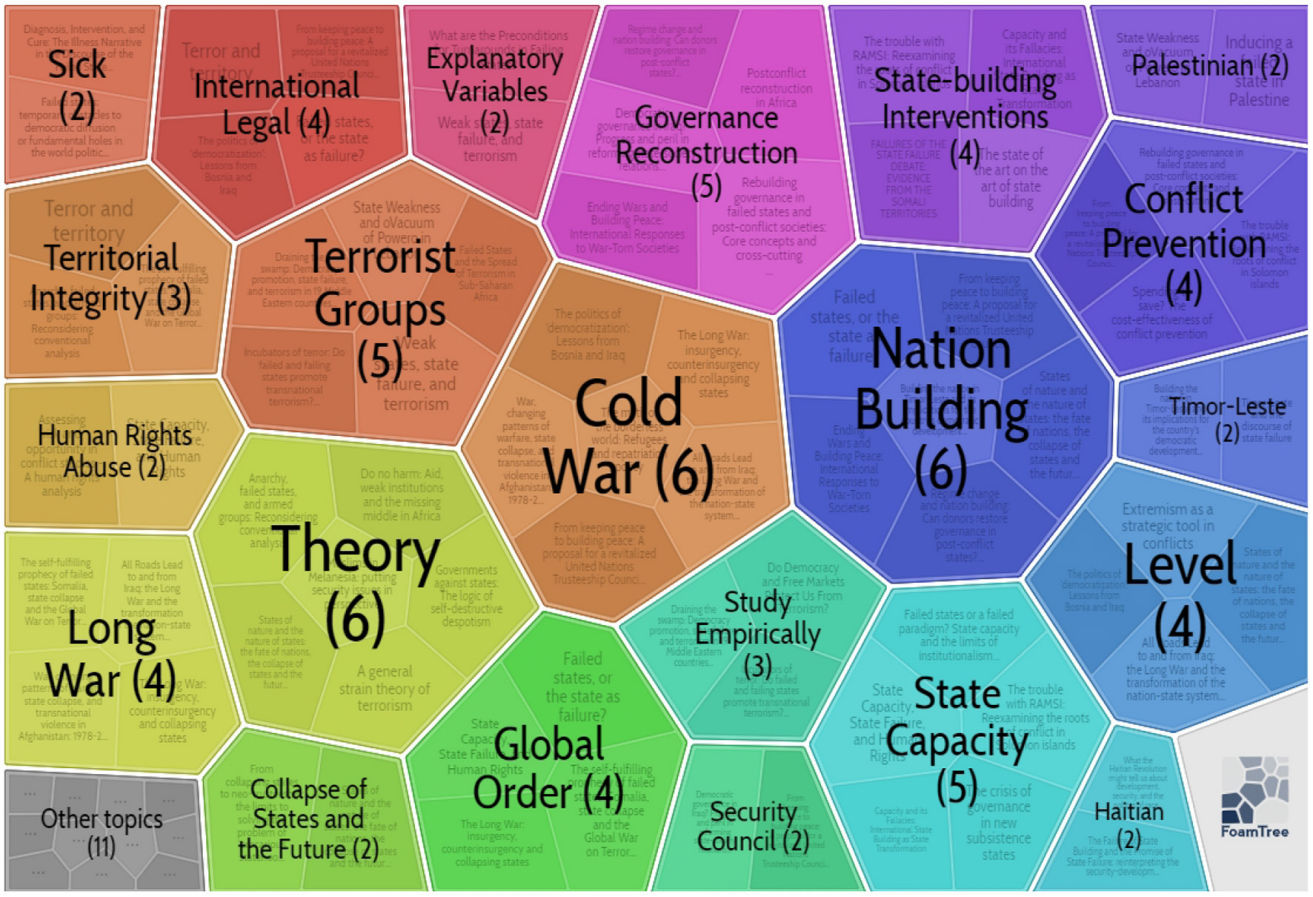
Keywords in Cluster 3 (by Carrot2 4.3.1).

The author applied the Carrot2 tool to explore each cluster further, employing the treemap methods to obtain more insight. First, the author used CiteSpace to get all clusters and save the cluster information in Carrot2 format by CiteSpace. Then the author uploaded the cluster information to the Carrot2 website to get the treemap by Carrot2, as we can see in Figures 7–**10**.

#### Cluster 0: State Fragility

Cluster 0 displays the largest cluster, involving 51 references whose mean published year is 2009. Cluster 0 has a silhouette score of 0.831, proving that this cluster is very consistent. Given that cluster 0 is the vast cluster, its theme is comparatively dispersed. Table S9 in the Supplementary Material shows more details. Carrot2 can process vital concepts derived from cited articles' titles, keywords, as well as abstracts using an algorithm method.

The first is the keywords. As is shown in Figure 7, Figure S14, and Table S9 in the Supplementary Material, the basic concepts of cluster 0 show significant discoveries connecting failed states (45), state failure (38) as well as state capacity (35), as seen in Figure 7's foam tree visualization. More details can be found in Table S9 in the Supplementary Material. Concepts like “state-building,” “political power,” “the rule of law,” “developing world,” for example, highlight this cluster's primary focus.

The second is the most frequent references. The frequency with which key references are cited shows these studies' relative relevance and contributions to failed/fragile/collapsed state research. As a result, recognizing this significant progress will help future researchers grasp the critical studies in failed/fragile/collapsed states (Li et al., 2017). Report about conflict, security, and development published by World Bank is the most cited reference in cluster 0 (World Bank, 2011). Ghani and Lockhart created a new research paradigm for failed/fragile/collapsed state studies, which is the second most cited reference (Ghani and Lockhart, 2009). The third most referenced article by Hagmann and Hoehne is on the multiple state-building processes and kinds of statehood that have arisen within Somalia (Hagmann and Hoehne, 2009).

The third is the vital literature from previous years. The timeline view depicts three stages of cluster 0's evolution (Figure S14 in the Supplementary Material). The first period was from 2005 to 2009. The first reference in cluster 0 provides an approach to the most contentious issues around globalization—scientific research, neoliberalism, governance—from the perspective of the “anthropological” problems (Hannerz, 2006). The first citation exploration during the first period is the article that provided relief in crisis times and built capacity in developing states to accelerate their advancement (Patrick, 2007). Rice and Patrick presented the Developing World's Index of State Weakness, which includes all 141 developing countries in four critical spheres: social welfare, security, political, and economic (Rice and Patrick, 2008). It is the second citation exploration during the first period. The second period was from 2010 to 2011. As the author has previously described, the enormous citation exploration among all clusters appeared. The third period was from 2012 to 2014. The most cited publication was published by Grävingholt et al. (2012), with no evidence of a citation exploration during this period. The following paragraph reviews some other high-frequency articles worth noticing.

More specifically, Taylor and Botea (2008) explained diversity in nation efficiency among the developing world's most war-prone countries. They discovered that the Vietnam war helped state-building. In comparison, the war in Afghanistan has been state-damaging. Goldstone et al. (2010) studied political uncertainty in states worldwide and built up a model that identified states that encountered instability. Acemoglu and Robinson (2012) convincingly proved that human-made political and economic systems determine economic prosperity (or scarcity of it). The distinctions between the Koreas are owed to the government that made these utterly different institutional paths. Paris (2010) distinguished between reasonable and unjustified critiques, calling for a more unified debate about the liberal peacebuilding's flaws and prospects.

#### Cluster 1: Successful Intervention

As shown in Figure 8, Table S10, and Figure S15 in the Supplementary Material, the second-largest cluster is cluster 1, comprising 27 publications over 9 years from 2010 to 2019. The author used Carrot 2 once more to in-depth analyse cluster 1.

The first is the keywords. According to the foam tree imagery, cluster 1's basic concepts include “developing world,” “aid effectiveness,” “civil war,” “failed states,” “service provision,” as well as “public health.”

The second is the most frequent references. Figure S15 in the Supplementary Material presents that this cluster is uneventful in citation frequency and exploration importance, despite high-profile references. The half-life metric shows how long it would take for half of the existing publications to become obsolete. The half-life of classic publications is greater than other forms of publications (Burton and Kebler, 1960).

The third is the vital literature from previous years. Both Menkhaus (2014) and Grävingholt et al. (2015) have a half-life score of 4.5, suggesting that these two classic studies contribute actively to cluster 1 study assessment. Menkhaus (2014) alleged that some remarkable successes had appeared at the local level in Somalia, both with formal and informal authority structures. Fragile statehood, according to Grävingholt et al. (2015), is described as failures in one or several of the state's core capabilities: governance, efficiency, and authority. The following paragraph discusses some other high-frequency articles worth noticing. Why poor states are recommended to diminish administrative autonomy while high-income entities are asked to reinforce it was analyzed by Fukuyama (2013). Gisselquist (2014) paid specific consideration to the character of foreign aiding, providing unique traction on concept progress on state-building. Lee et al. (2014) discovered an extraordinarily limited manifest in a steady relation between statehood and supply distribution. Pettersson and Wallensteen (2015) confirmed that although the peace negotiations expansion been part of a constructive tendency since 2011, several peace processes remained weak by the end of 2014.

#### Cluster 2: Peace Research

The first is the keywords. As seen in Figure 9, Figure S16 and Table S11 in the Supplementary Material, the third-largest cluster was cluster 2, comprising 68 references over 9 years from 2008 to 2017. Cluster 2 had a silhouette value of 0.913. Carrot2 was used once more to delve further into cluster 2. According to the foam tree visualization, cluster 2's key principles include “state failure,” “national security,” “growing,” “security forces,” “security governance,” “citizens,” “foreign aid, militias,” “risk assessment,” and “the Democratic Republic of Congo”. Figure S16 shows that this cluster was considerable in citation frequency and explorations importance with high-profile references.

The second is the vital literature from previous years. Hendrix (2010) determines and addresses key theoretical and assessment issues supported by measures of state competence in investigations of civil conflict. He proposed three factors to describe state capacity: the first, rational validity, which encompasses administrative competence; the second, rentier-autocraticness; and the last, neo-patrimoniality (Hendrix, 2010). From Syria to Sudan, authorities maintain tacit links with militias that utilize terrorism against opposing groups and communities (Carey et al., 2015). According to Carey et al. (2015), certain regimes may dodge responsibility for violence and repression by delegating violence to these informal state–militia formations. Cohen and Nordås (2015) discover that governments use sexual violence as a supplement to militia-based violence. Militias that have recruited youngsters are also linked to greater rates of sexual violence, according to the researchers. According to Coggins (2015), most failed, and failing nations are not prone to terrorism. Those at war or undergoing governmental collapse are substantially more prone to experience and cause fear among the “most unsuccessful” regimes.

More specifically, the number of institutions in the organization, the level of centralization among these institutions, and the division of power across them are all considered by Bakke et al. (2012) to be three essential characteristics of fragmentation. Chandler (2012) suggested that human security can be conceptually analyzed in terms of post-intervention. Clunan and Harold (2010) investigate whether and how “ungoverned areas” contribute to global instability, considering the many locations where state authority is challenged. They proposed that the most important ambition of all state-construction is to establish a justifiable state by the people it governs. Lake (2016) discusses the crucial dilemma between validity and loyalty that all international state-building efforts face, such as the well-known success stories of West Germany and Japan after 1945. Kreutz (2010) gives fresh intelligence on the break and end dates of armed conflicts, as well as the methods of ending them. His findings showed that following government triumphs or the deployment of peacekeepers, intrastate wars are less likely to repeat. Waal (2009) looked at how unstable African nations functioned politically and economically. He argued that the leaders in these countries use the lens of a ‘political marketplace' to obtain the highest reward for loyalty within patrimonial systems, including Sudan and the DR Congo. Horizontal imbalances between politically significant ethnic groups and governments, according to Cederman et al. (2011), may increase ethnonationalist conflict. They also discovered that in countries with extreme inequality, both affluent and poor groups fight more often.

#### Cluster 3: Failed State

As seen in Figure 10, Figure S17, and Table S12 in the Supplementary Material, the fourth largest cluster was cluster 3, comprising 62 references over 7 years from 2000 to 2007. Cluster 3 had a silhouette value of 0.934. Carrot2 was used once more to delve further into cluster 3.

The first is the keywords. According to the foam tree visualization, cluster 3's key principles include “Cold War,” “nation-building,” “terrorist groups,” “long war,” “state capacity,” “global order,” “territorial integrity,” “conflict prevention,” “international legal,” “security council,” and “global order”. Figure S17 in Supplementary Material shows that this cluster was considerable in citation frequency and explorations importance with high-profile references.

The second is the vital literature from previous years. More specifically, Fearon and Laitin (2004) argued that, ironically, the Bush administration had undertaken state-building projects. They stated that the world was evolving in such a manner that the most excellent security dangers and difficulties now stem from the repercussions of political turmoil, humiliation, and misrule in the third world, rather than from superpower security rivalries such as China and Russia (Fearon and Laitin, 2004). Rotberg (2004) proposes a novel theory that defines and categorizes situations on a spectrum ranging from weak to failed to collapsed. Depth case studies of countries that have collapsed and dissolved are used to demonstrate the state failure paradigm (Somalia, Sierra Leone, the DR Congo, Sudan), states that are dangerously weak (Tajikistan, Sri Lanka, Indonesia), and states that are safe although weak (Tajikistan, Sri Lanka, Indonesia, Colombia,). Over 10 million people have died in failed governments' civil conflicts since 1990, and hundreds of millions more have been deprived of basic rights (Rotberg, 2010). Terrorism has only exacerbated failing governments. Rotberg (2010) looked at how and why states degrade, as well as what may be done to prevent them from collapsing. According to political, social, and economic factors, he defined and classified strong, weak, failing, and collapsed nation-states (Rotberg, 2010). Better domestic government in poorly governed, failing, and occupied polities, according to Krasner (2004), would need the transcendence of recognized standards, including the development of shared sovereignty in unique regions.

More precisely, Piazza revealed that the severity of state failures in the Middle East equals the intensity of terrorist attacks, providing an empirical basis for the link between state failure and terrorism (Piazza, 2007). According to Rotberg, there are many revealing signposts along the path to state collapse (Rotberg, 2002). On the economic front, living conditions steadily deteriorate as elites reward favored families, clans, or small groups with cash benefits. Leaders and their cronies violate democratic norms on the political level. On a personal level, damaging actions made by individuals have nearly invariably resulted in state failure. President Mobutu Sese Seko of Zaire, for example.

The likelihood of effective US-led state-building in the Arab World and beyond, according to Berger and Weber (2006), is the most restricted they have ever been. The terrorist acts on September 11, 2001, according to Hagel (2004), were harbingers of a turning moment in US history. Ismail (2016) developed a theoretical foundation for the study of state fragility phenomenon by applying the contract method of state theory, which emphasizes structural circumstances as the fundamental cause of state failure and state fragility. He claimed that the failure of certain post-colonial civilizations is because of their inherent social fragility. According to Li (2005), democratic engagement in a country minimizes transnational terrorist events. Additional explanatory factors need to be found, according to Newman (2007), since weak or failing nations may offer an enabling environment for particular sorts of terrorist organizations to operate.

Finally, the danger of terrorism, which has flared up in Indonesia and Africa, has brought failed nations to a new level of urgency and significance (Rotberg, 2004). Failure used to be purely humanitarian, with more minor consequences for peace and security (Rotberg, 2004). Nowadays, scholars are more concerned about the relationship between failed states and the international system.

### Roadmap for Future Failed/Fragile/Collapsed States Research

The author identifies four under-explored topics in failed/fragile/collapsed state studies based on the thorough evaluation of failed/fragile/collapsed state studies offered in the preceding sections. The author also proposed some appropriate challenges for future research on each of the topics, as noted below:

Q1. How does foreign aid help failed/fragile/collapsed states and promote economic growth? What conditions are foreign aid not helpful to the failed/fragile/collapsed states' recovery and humanization disaster alleviation?
Q2. How will international intervention play its positive role in helping failed/fragile/collapsed states start-up their economy and minimize civil conflicts triggered by guerrilla and rebels? How can the potential adverse effects of international intervention play in failed/fragile/collapsed states be effectively avoided?

Second, a failed/fragile/collapsed state is not an island but is firmly anchored in the international societies and neighboring countries' nets. The best explanation for why nation-states failed can be in the basic system of the international environment that causes it. Although Jörgensen et al. (2011) emphasized the multi-level embodiment of failed/fragile/collapsed states, only some case studies looked at the multi-level international elements' effect on states' fragilities. There is limited experimental research, to the best of our understanding, which comprehensively examines the embodiment of failed/fragile/collapsed states at the macro, meso, and micro levels simultaneously. The international systems, neighboring countries' environmental backgrounds, social and political, and institutional factors can influence state failure or fragility. Given that failed/fragile/collapsed states are embedded in external nations, institutes, and racial backgrounds that can constrain and enable state fragility (Siqueira, 2014), future research should develop a coordinated research structure to illustrate how state fragility is rooted in the macro, meso, and micro environment holistically. Some issues that need consideration include:

Q3. What role does failed/fragile/collapsed states play in the macro, meso, and microenvironment? What are the geological effects of the ethical and communal background of states' fragilities?

Finally, recent studies have concentrated on the consequences of the United States' emergence as a “failed state” during the Trump administration for his catastrophic way to respond to Covid-19 (Nowroozpoor et al., 2020), West-Europe's welfare burden states (Meuleman and Delespaul, 2020), PRC (People's Republic of China) as an authoritarian state with ironically high legitimacy (Nathan, 2017). It is controversial that these countries be defined as failed states or due to scholars' habit of being prudent and pessimistic in academic thinking. The author calls for further research to focus on some more in-depth country studies on how they recovered from failure or collapsing status, for example, Rwanda's economic miracle after the genocide (Zorbas, 2004), Uganda's successful AIDS eradication case after the topple of Amin autocracy (Parkhurst, 2001). Interesting questions include:

Q4. To what degree do racial or economic considerations interact for circumstantial dynamics that contribute to state collapse or fragility?
Q5. How do developed and developing countries vary in the configurations resulting in state failure/fragility?
Q6. What factors prompt some fragile states to recover from failure and pick up economic growth and racial reconciliation?
Q7. What are the challenges other fragile/failed states face when they launch economic and democracy recovery programs? What are their strategies for conquering these challenges?

## Limitations

There are some limitations to this review.

First, it is a pity that this review only includes the 2,417 SSCI documents, and this may trigger imbalances since the 38,835 secondary documents cited by these SSCI papers are much more numerous than the primary documents. Another shortcoming is that the 38,835 secondary documents cited are not included in the proposed analyses above. The research depends on the search strategy defined in Results Section. Utilizing different search phrases, searching by keywords, abstract, and title, or searching through a dataset rather than the WoS database will affect the number of articles identified and, as a result, the performance. Despite being one of the most extensive significant abstracts and citation collections of peer-reviewed papers, the WoS Core Collection is not without flaws; it may not include all studies. As a result, other databases, particularly the growing number of preprints accessible on Google Scholar and Scopus, might have contributed additional insights not accessible in this research domain. However, since the emphasis of this research was on detecting the fundamental structure rather than counting citations or co-citations, this problem was mitigated. This article's findings are still crucial to understanding the landscape and evolution of fragile/failed/collapsed states studies among political science, economics, and political economics.

Second, another limitation and criticism of this review relate to the method, which includes articles and reviews. Some criticisms need to be prioritized, which relates to the methodology for building the corpus and the inclusion of reviews in addition to articles (McMahan and McFarland, 2021). It would be helpful to try coupling analysis of articles only. Including reviews in order to carry out a new review is controversial in academic circles. First, papers cited by review articles may experience a significant drop in future citations, as McMahan and McFarland (2021) suggested. They looked at the impact of review articles on the publications they referred to and discovered that works cited in formal review articles lose a significant number of future citations. Rather than the individual publications referenced in the review, the review is often cited. In brief, reviews are a sort of creative destruction in that they establish a cohesive sub-domain based on a set of exemplars and reduce the impact of non-exemplars in the future (McMahan and McFarland, 2021). Although, it seems to be a convention in bibliometric analysis to analyze articles and reviews together (Deng et al., 2020; Yang et al., 2020; Ye N. et al., 2020; Ye P. et al., 2020; Yeung and Mozos, 2020; Yu et al., 2020; Zhai et al., 2020). My review is not exempt, which uses a joint analysis of articles and reviews, but it should not be overlooked that the analysis might be more scientific and rational if the reviews had been removed from the search query.

Ultimately, the restriction on English-language documents and the paper type restriction on articles or reviews may lead to research blind spots. It is debatable if citations should be used as a substitute for the importance of scientific contributions (Garfield, 1979; Fong and Wilhite, 2017). In scientometric analyzes, citation indices, including the cumulative number of citations, are often criticized for calculating influence and recognizing trends. Since citations take years to gain, relying on multiple citations can reduce specific important patterns, particularly in more recent research.

## Conclusions

This bibliometric review of failed/fragile/collapsed states research helps practitioners understand this field and provides necessary implications. Based on the bibliometric analysis using science mapping approaches, this review's contribution is based on a not pre-selected and better objective examination of the basic framework as well as the progression of failed/fragile/collapsed state studies. Previous studies in this domain have specialized in a single topic or strong-influential journals in a specialized field while overlooking trends as well as fundamental studies from new journals and disciplines. This bibliometric analysis concentrated on thousands of reference statistics other than a limited total of papers pre-chosen by the analyst. It is statistics-based and less bias-oriented than previous analyzes. Reviews may influence the future direction of study in an emerging research topic by combining the results into a cohesive narrative. The distinctive discursive tendency of reviews, which focuses on novices' clarity of synthesis from a perspective wholly engaged in current academia, suggests that they might play a generative role in research output creation (McMahan and McFarland, 2021).

This bibliometric analysis makes three donations. First, this review provides a unique prospect in failed/fragile/collapsed state studies through a detailed, methodical, and objective analysis. The initial qualitative reviews have traditionally relied on personal judgment, whereas only a few quantitative review studies have focused solely on statistical evidence. This review enhances earlier articles by doing co-citation and co-occurrence structure analysis and envisioning them through a detailed, precise mechanism.

For starters, due to government mismanagement and corruption, failed/fragile/collapsed states often have difficulty securing funding from self-enriching bureaucracies' hands (Khan, 2015). Although some scholars have challenged the efficacy of foreign humanitarian aid in improving government performance in developing countries, especially failed/fragile/collapsed states (Waheed, 2014). Exploring the boundary conditions of foreign humanitarian aid's positive effects in helping failed/fragile/collapsed states may become increasingly relevant in the future, and more research on the negative impact of foreign humanitarian aid on failed/fragile/collapsed states is called for. Research into the trends and internal processes that contribute to this negative impact and how to mitigate it may have practical suggestions for politics, principally in the least developed countries, who wish to rebuild their failed/fragile/collapsing motherland and reduce humanitarian disasters by encouraging their countries to develop economies. The following are some of the provocative questions.

Secondly, current research has focused primarily on the failure/fragility states' political and economic consequences, with little attention paid to their interdependencies. Understanding their dynamic interaction could be crucial in understanding how failed/fragile/collapsed states emerge. It is possible there is not only one “ideal model” for complicated failed/fragile/collapsed states. As long as the failed/fragile/collapsed states and the environment “match,” different failed/fragile/collapsed states will emerge by circumstances in different situations. To solve this challenge, future studies could look at different combinations of cross-cultural social considerations and supporting external factors at the local, provincial, and country level. Rather than pursuing once-in-a-lifetime strategies, policymakers and economists can seek context-unique solutions. The following questions were given to us.

Finally, the results show articles in political and economic journals are the most referred to in this field, barring new competence from other subjects from entering the fragile/failed/collapsed state studies. As a result, fragile/failed/collapsed state research journals can broaden their scope and incorporate expertise from multiple disciplines.

## Author Contributions

The author confirms being the sole contributor of this work and has approved it for publication.

## Conflict of Interest

The author declares that the research was conducted in the absence of any commercial or financial relationships that could be construed as a potential conflict of interest.

## Publisher's Note

All claims expressed in this article are solely those of the authors and do not necessarily represent those of their affiliated organizations, or those of the publisher, the editors and the reviewers. Any product that may be evaluated in this article, or claim that may be made by its manufacturer, is not guaranteed or endorsed by the publisher.
