# Supplementary figures and images for: The Past, the Present, and the Future: A Bibliometric Analysis of Failed/Fragile/Collapsed State Research During 1990–2020
Source: Front Res Metr Anal. 2022 Feb 4;7:720882. doi: 10.3389/frma.2022.720882 (PMC8855057; doi:10.3389/frma.2022.720882)

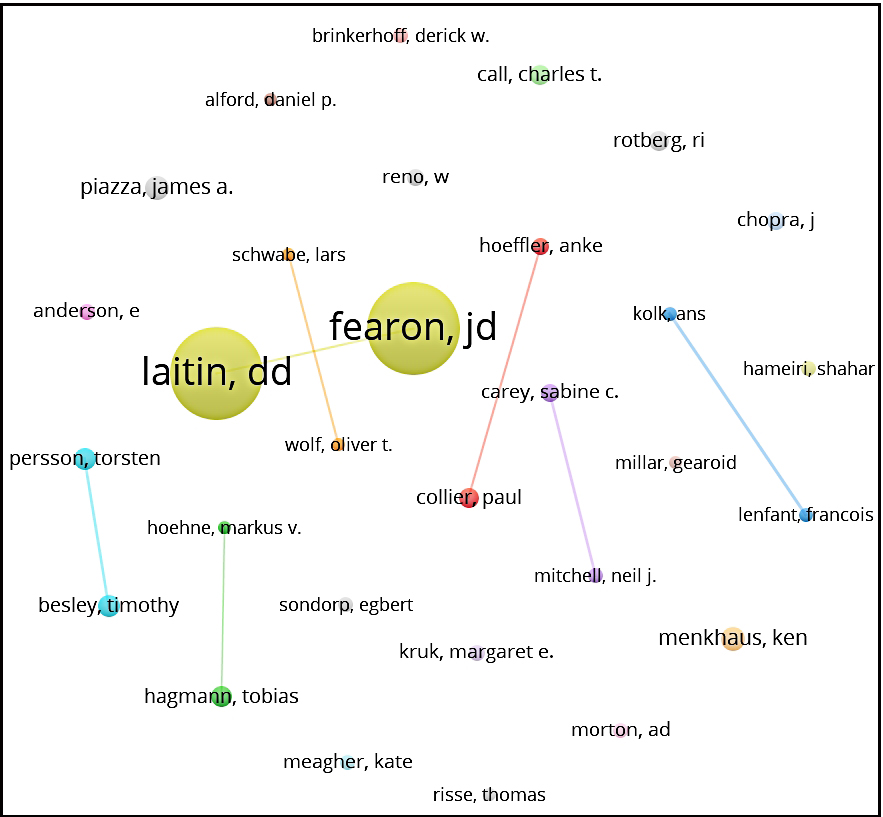

Supplement: Supplementary file 2 [file Data_Sheet_1.ZIP › online appendix figures/appendix figure A10.jpg]

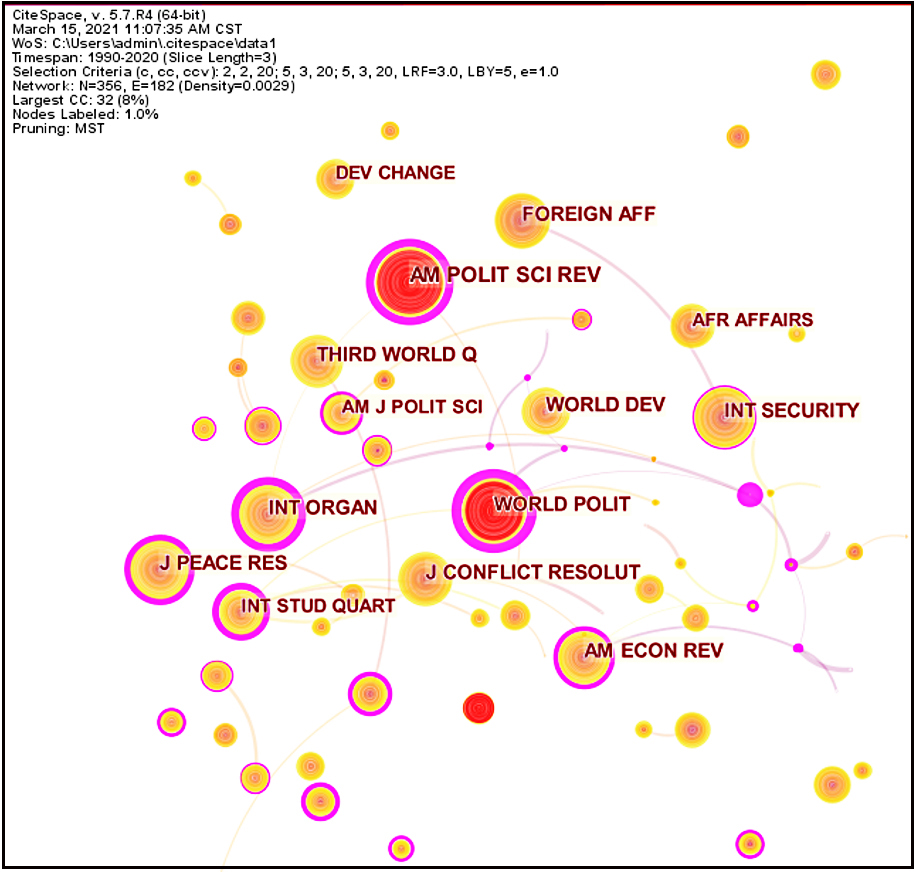

Supplement: Supplementary file 2 [file Data_Sheet_1.ZIP › online appendix figures/appendix figure A11.jpg]

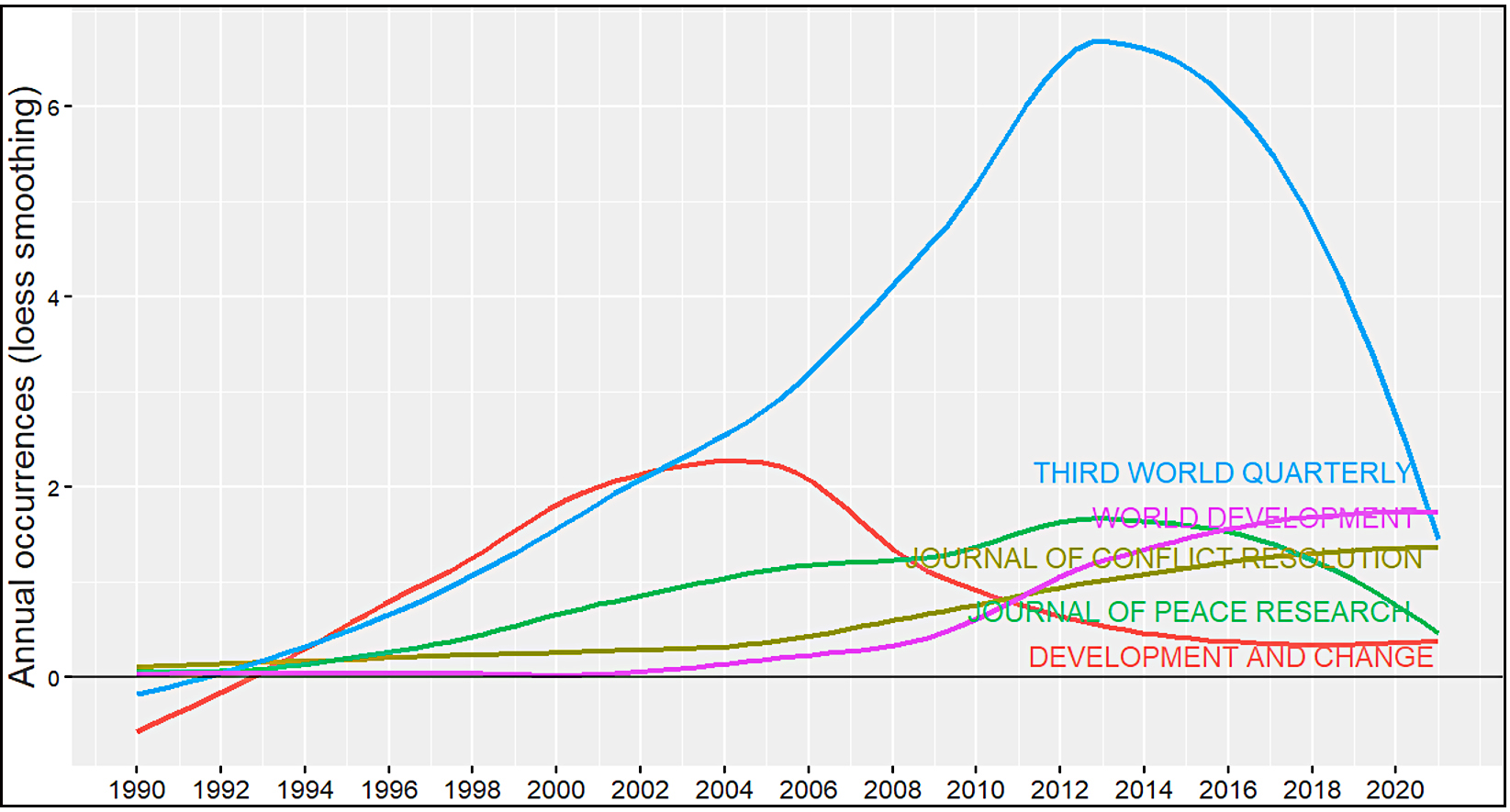

Supplement: Supplementary file 2 [file Data_Sheet_1.ZIP › online appendix figures/appendix figure A12.jpg]

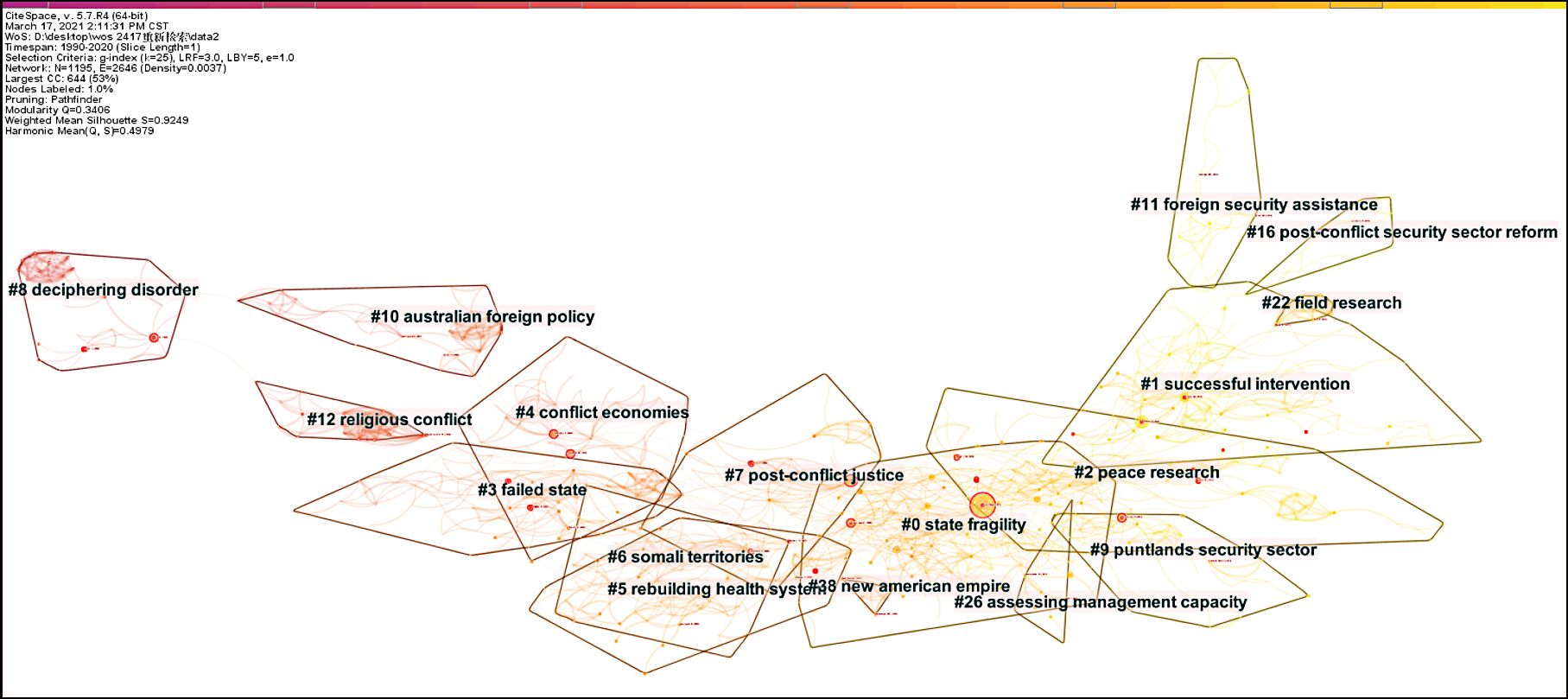

Supplement: Supplementary file 2 [file Data_Sheet_1.ZIP › online appendix figures/appendix figure A13.jpg]

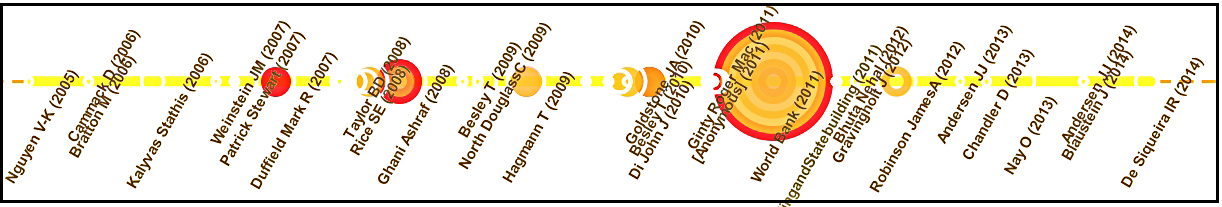

Supplement: Supplementary file 2 [file Data_Sheet_1.ZIP › online appendix figures/appendix figure A14.jpg]

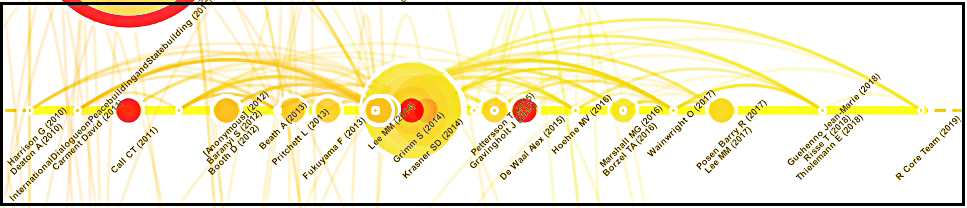

Supplement: Supplementary file 2 [file Data_Sheet_1.ZIP › online appendix figures/appendix figure A15.jpg]

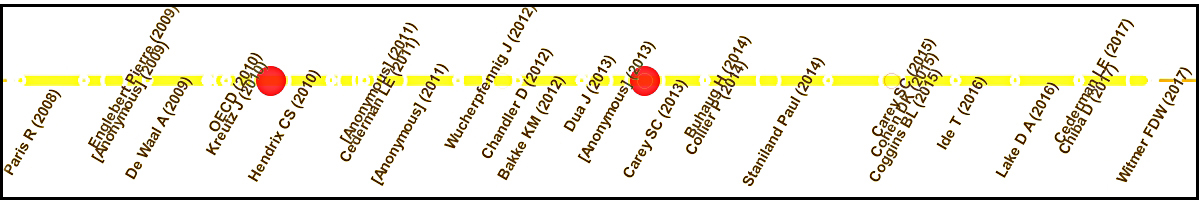

Supplement: Supplementary file 2 [file Data_Sheet_1.ZIP › online appendix figures/appendix figure A16.jpg]

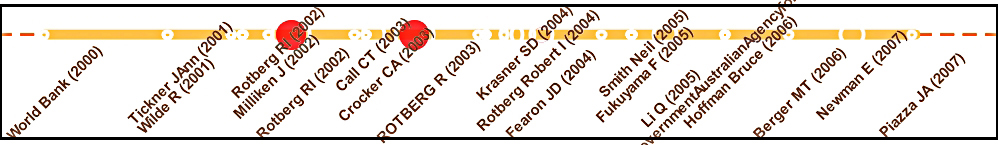

Supplement: Supplementary file 2 [file Data_Sheet_1.ZIP › online appendix figures/appendix figure A17.jpg]

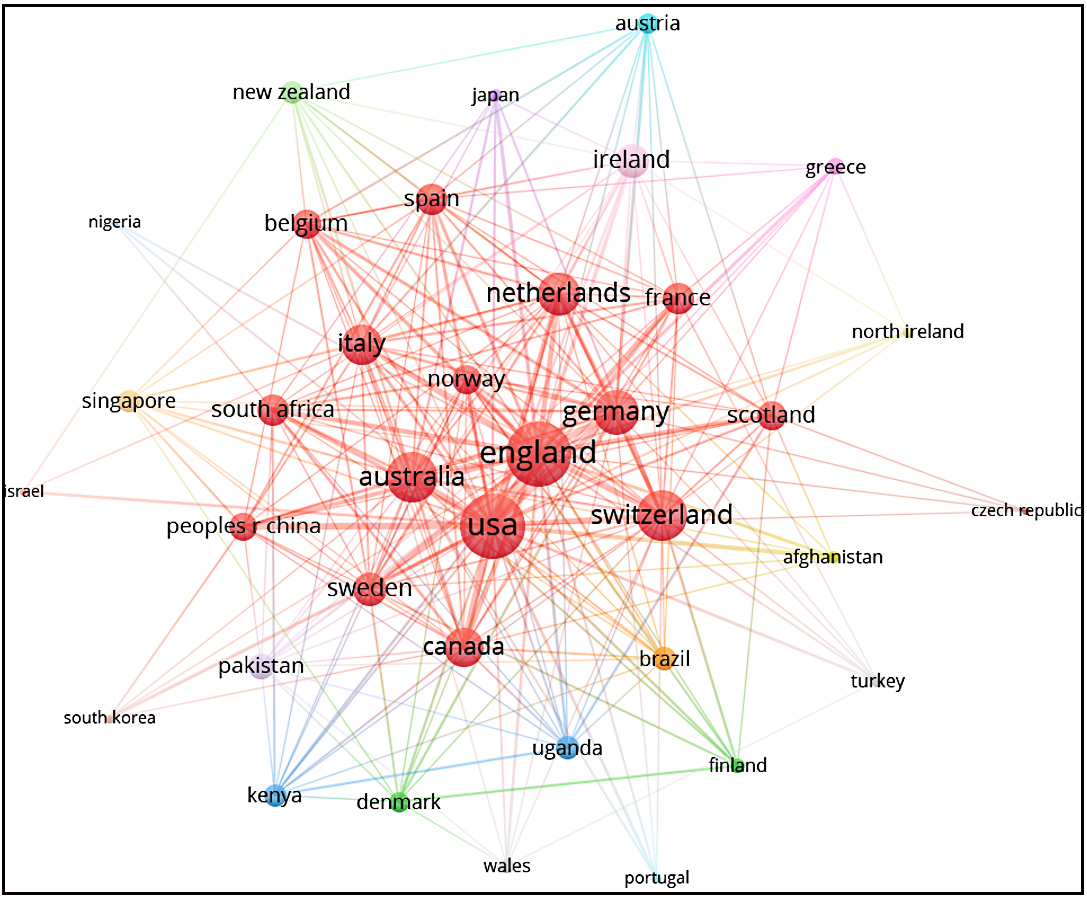

Supplement: Supplementary file 2 [file Data_Sheet_1.ZIP › online appendix figures/appendix figure A5.jpg]

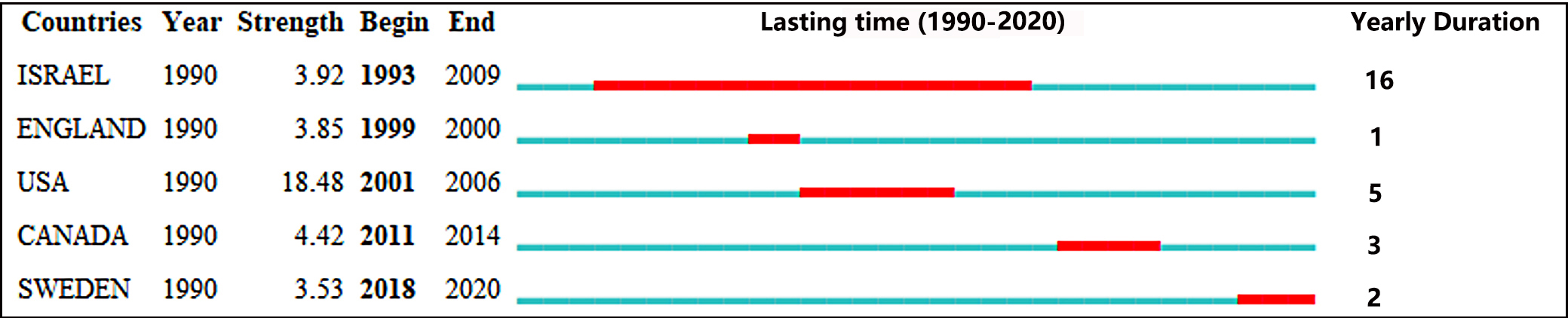

Supplement: Supplementary file 2 [file Data_Sheet_1.ZIP › online appendix figures/appendix figure A6.jpg]

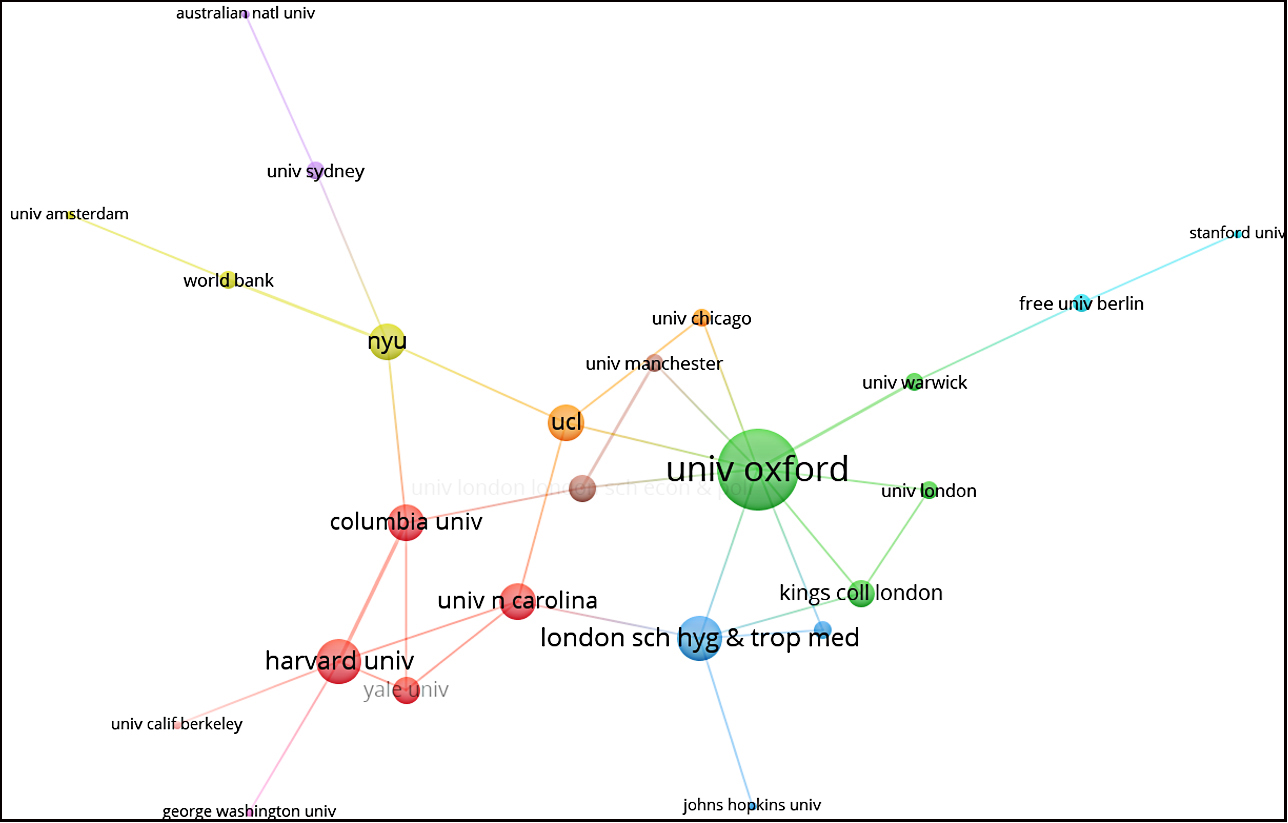

Supplement: Supplementary file 2 [file Data_Sheet_1.ZIP › online appendix figures/appendix figure A7.jpg]

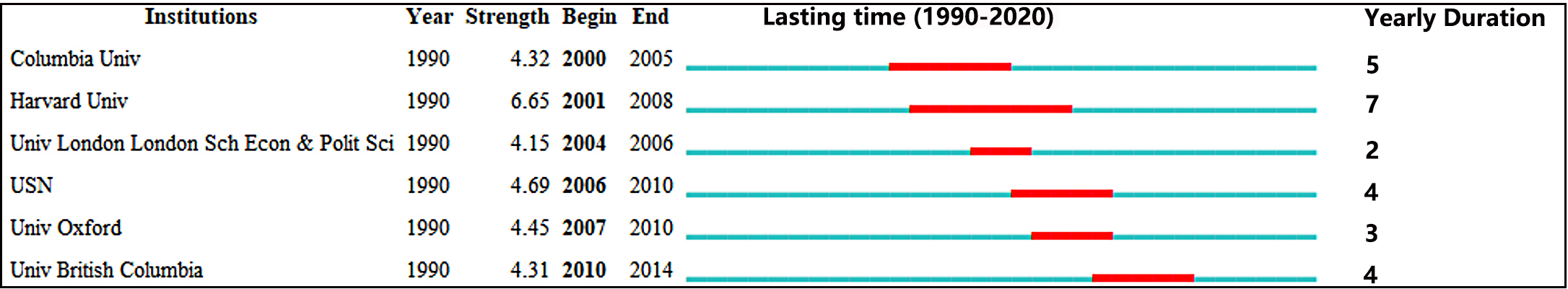

Supplement: Supplementary file 2 [file Data_Sheet_1.ZIP › online appendix figures/appendix figure A8.jpg]

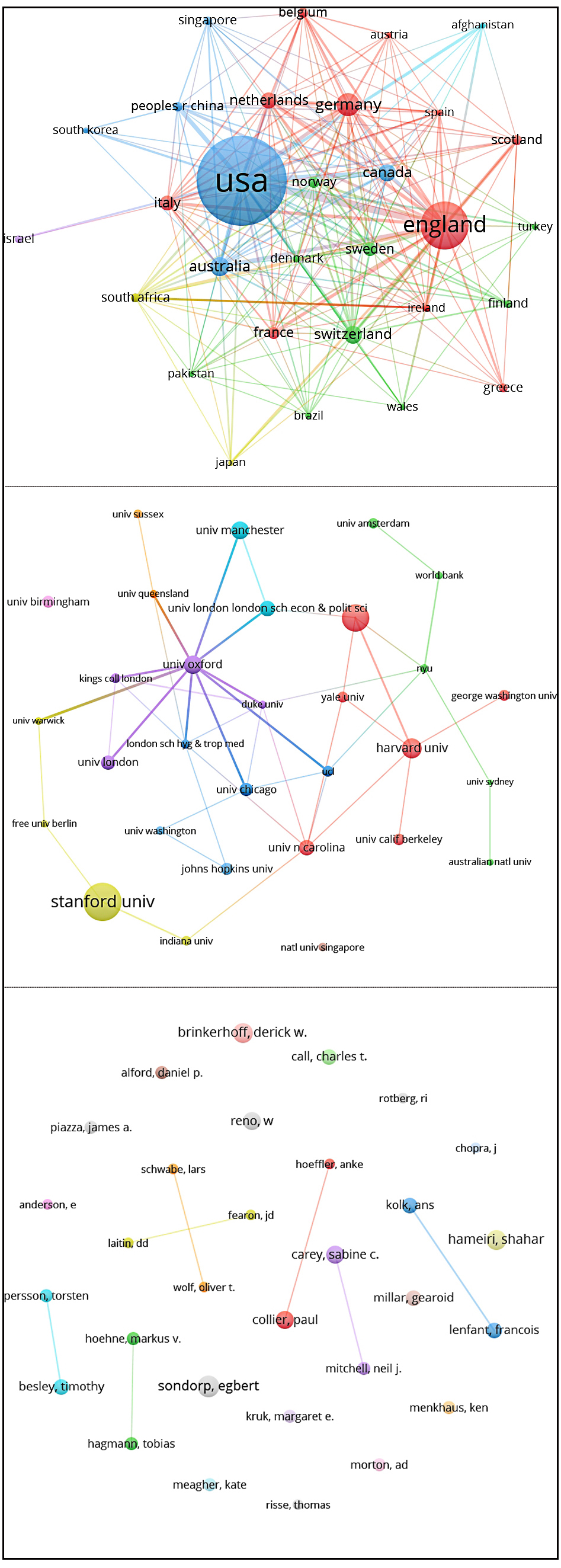

Supplement: Supplementary file 2 [file Data_Sheet_1.ZIP › online appendix figures/appendix figure A9.jpg]

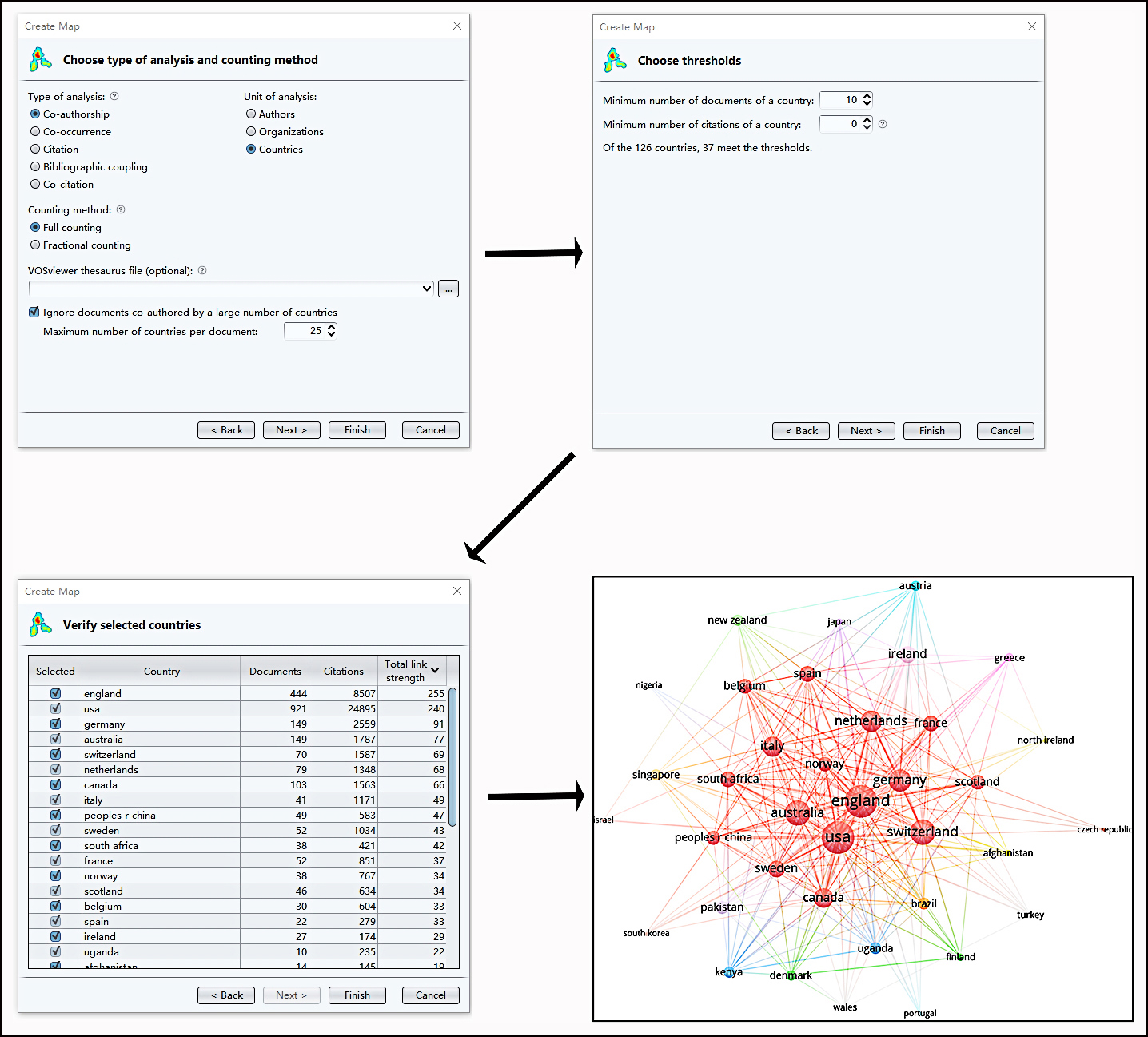

Supplement: Supplementary file 2 [file Data_Sheet_1.ZIP › online appendix figures/appendix figureA1.jpg]

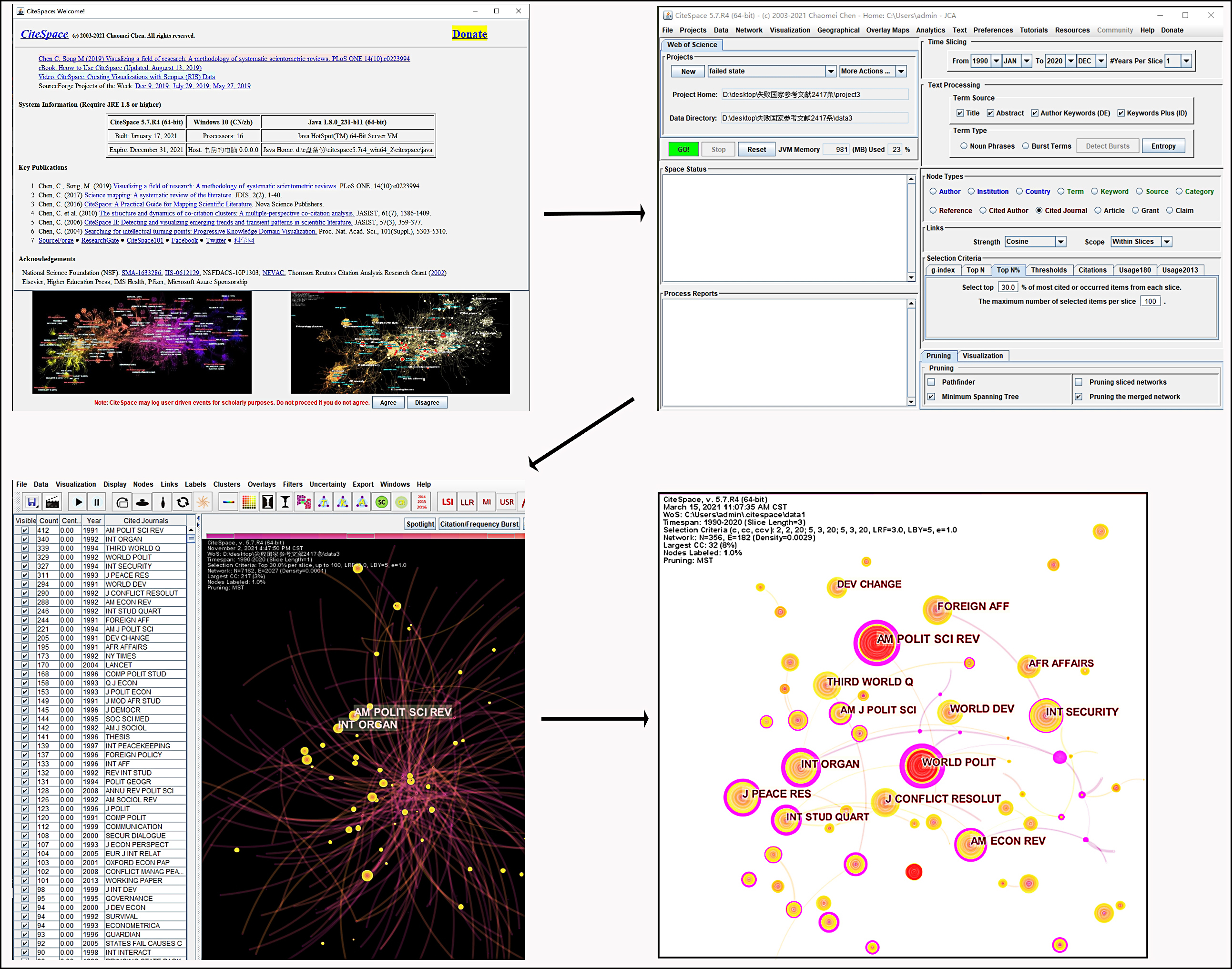

Supplement: Supplementary file 2 [file Data_Sheet_1.ZIP › online appendix figures/appendix figureA2.jpg]

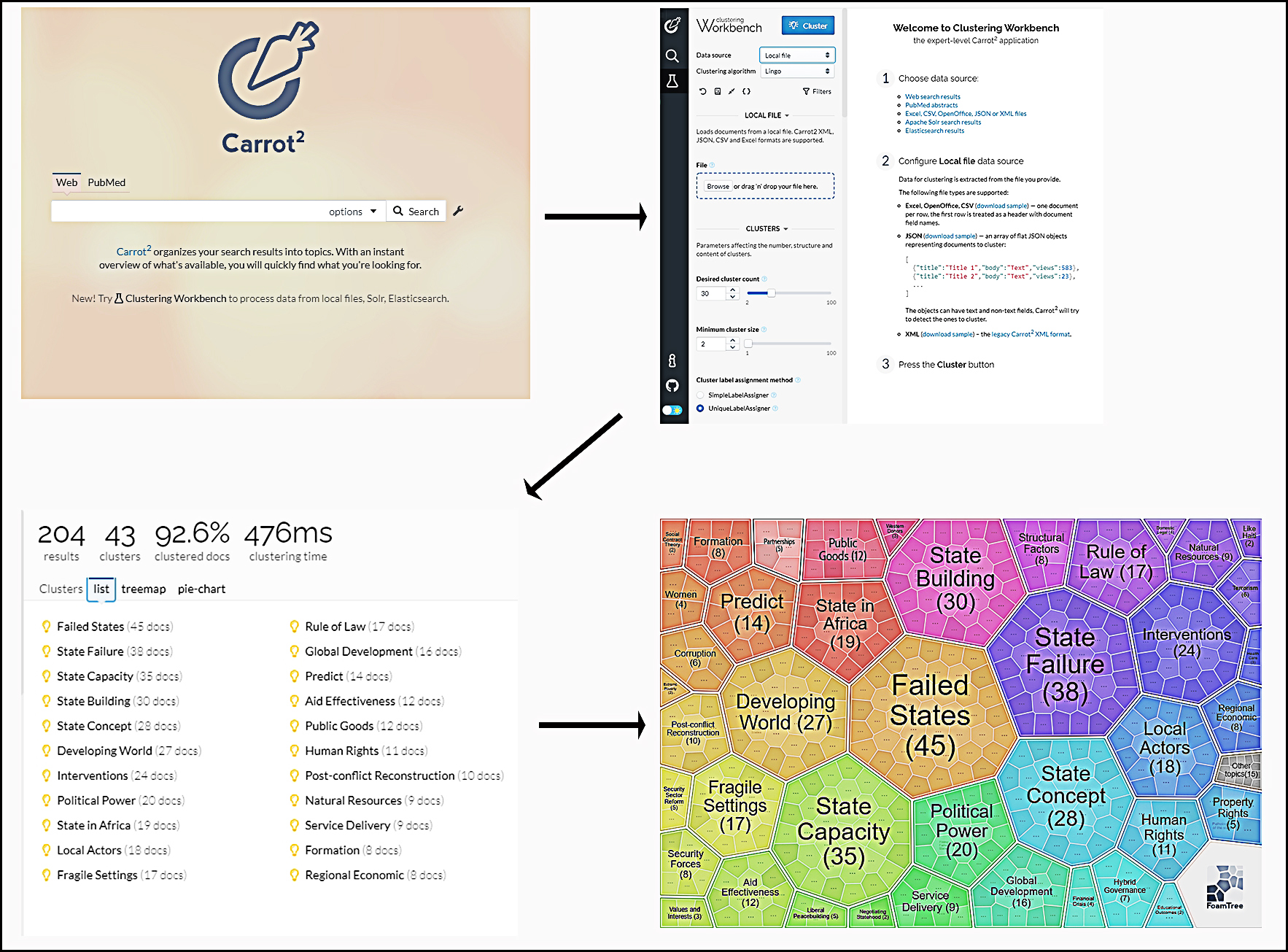

Supplement: Supplementary file 2 [file Data_Sheet_1.ZIP › online appendix figures/appendix figureA3.jpg]

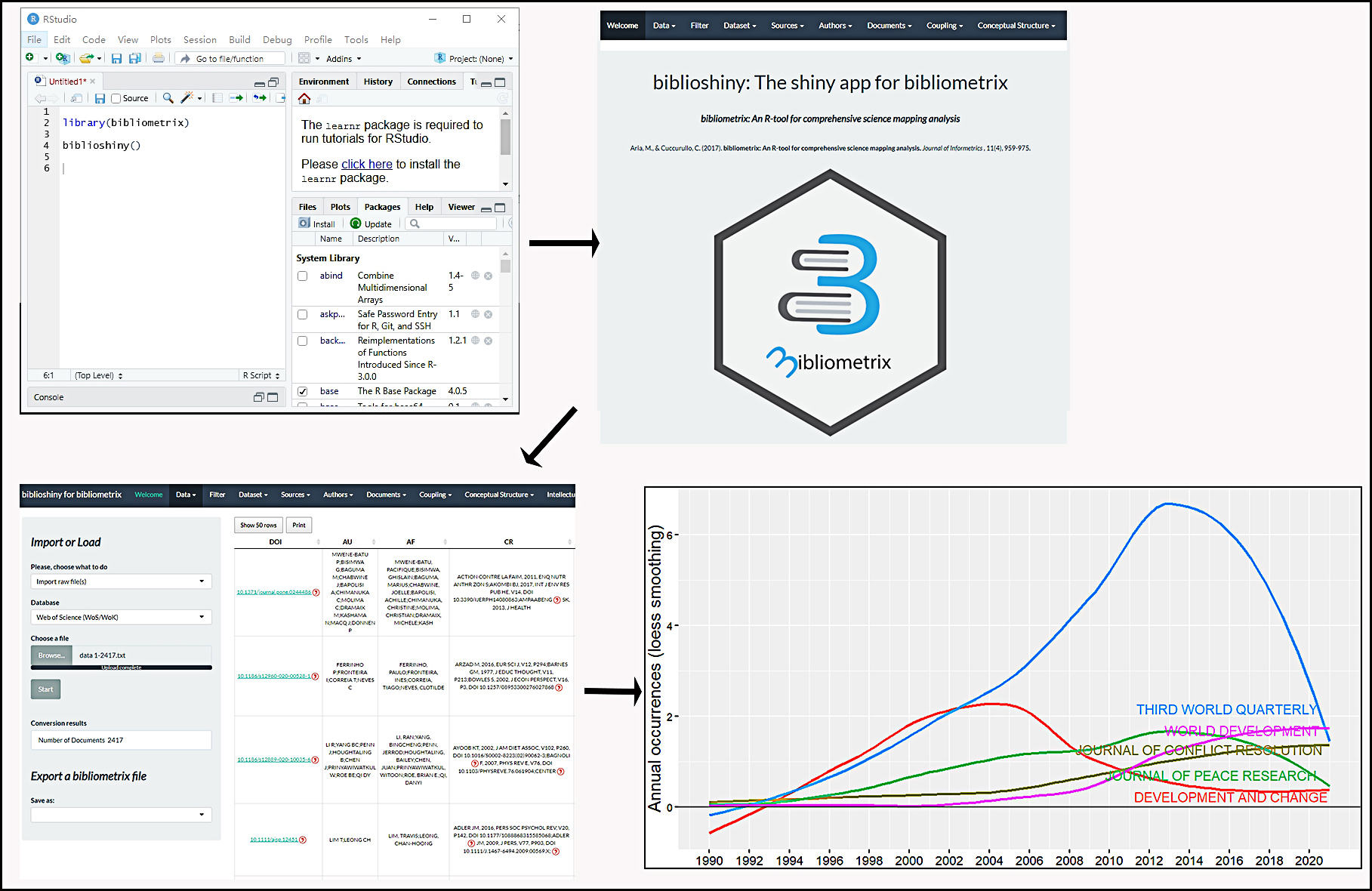

Supplement: Supplementary file 2 [file Data_Sheet_1.ZIP › online appendix figures/appendix figureA4.jpg]
